# Design, synthesis, and evaluation of the novel ozagrel–paeonol codrug with antiplatelet aggregation activities as a potent anti-stroke therapeutic agent

**DOI:** 10.3389/fphar.2024.1362857

**Published:** 2024-03-19

**Authors:** Chijing Zuo, Fulong Yan, Jie Wang, Yulong Zhu, Wenhui Luo, Yan Liu, Wanhui Liang, Weidong Yu, Jingwei Zhang, Daiyin Peng, Xiaodong Ma, Can Peng

**Affiliations:** ^1^ School of Pharmacy, Anhui University of Chinese Medicine, Hefei, China; ^2^ MOE-Anhui Joint Collaborative Innovation Center for Quality Improvement of Anhui Genuine Chinese Medicinal Materials, Hefei, China; ^3^ Generic Technology Research Center for Anhui TCM Industry, Anhui University of Chinese Medicine, Hefei, China; ^4^ Rural Revitalization Collaborative Technical Service Center of Anhui Province, Anhui University of Chinese Medicine, Hefei, China; ^5^ Anhui Province Key Laboratory of Pharmaceutical Preparation Technology and Application, Hefei, Anhui, China; ^6^ Center for Xin’an Medicine and Modernization of Traditional Chinese Medicine of IHM, Anhui University of Chinese Medicine, Hefei, China

**Keywords:** ozagrel, paeonol, antiplatelet aggregation activity, pharmacokinetic properties, neuroprotective effect, molecular docking

## Abstract

**Introduction:** Ischemic stroke is the second most common chronic disease worldwide and is associated with high morbidity and mortality. Thromboembolism and platelet aggregation are the most characteristic features of stroke. Other than aspirin, no standard, accepted, or effective treatment for acute ischemic stroke has been established. Consequently, it is essential to identify novel therapeutic compounds for this condition.

**Methods:** In this study, novel ozagrel/paeonol-containing codrugs were synthesized and characterized using ^1^H-NMR, ^13^C-NMR, and mass spectroscopy. Their antiplatelet aggregation activity was evaluated, with compound PNC_3_ found to exhibit the best effect. Subsequently, studies were conducted to assess its neuroprotective effect, pharmacokinetic properties and model its binding mode to P2Y12 and TXA2, two proteins critical for platelet aggregation.

**Results:** The results indicated that PNC3 has good bioavailability and exerts protective effects against oxygen-glucose deprivation injury in PC12 cells. Molecular docking analysis further demonstrated that the compound interacts with residues located in the active binding sites of the target proteins.

**Conclusion:** The codrugs synthesized in this study display promising pharmacological activities and have the potential for development as an oral formulation.

## 1 Introduction

Cardio-cerebrovascular diseases are the primary chronic diseases globally, posing a major hazard to human health (Amy Guzik et al., 2017; Tsao et al., 2023). Ischemic stroke (IS) is the most typical manifestation of cardio-cerebrovascular diseases, with its high rate of morbidity, disability, and mortality (Hankey, 2017). Most ISs are thromboembolic in origin. Currently, the pathogenesis of thromboembolism involves the following aspects: 1) vascular endothelial injury triggers platelet aggregation through the release of prostaglandins and coagulation factors (Hu et al., 2017; Shaik et al., 2021). 2) Abnormal hemorheology leads to red blood cell adhesion and platelet aggregation (Daniel et al., 2017). 3) Hyperlipidemia-induced elevation in low-density lipoprotein directly promotes platelet aggregation (Van Lammeren et al., 2012). 4) Platelets release TXA2, which induces changes in platelet morphology and subsequent aggregation (Mustard et al., 1980). Unfortunately, there is no reliable data about the effects of any other antiplatelet drugs apart from aspirin. Hence, efficient prevention of the formation of thromboembolism and aggregation of platelets is the focus of current medical research.

Ozagrel, also referred to as (E)-sodium 4-(1-imidazolomethyl) cinnamate, is the first commercially available inhibitor of thromboxane A_2_ (TXA_2_) synthase, which is highly selective and potent. It has been demonstrated to possess antiplatelet aggregation and vasodilatory effects (Ishitsuka et al., 2004; Ishitsuka et al., 2009). It has been used in the treatment of cerebral ischemic stroke with promising therapeutic outcomes (Zhang et al., 2013; Bhatia et al., 2021). Presently, ozagrel combined therapy is widely used in clinical practice, especially in combination with traditional Chinese medicine formulas or monomers (Park et al., 2015; Wada et al., 2016; Zhang and Zheng, 2021).

Paeonol (2′-hydroxy-4′-methoxyacetophenone), a bioactive ingredient in the Cortex Moutan of traditional Chinese medicine, has been found to inhibit antiplatelet aggregation and anti-inflammatory activity and suppress oxidative stress (Zhang et al., 2019). Numerous studies have demonstrated that paeonol can protect the brain from damage and reduce the area of cerebral infarction caused by ischemic stroke (Zhao et al., 2018; Wu et al., 2023). Nevertheless, paeonol has poor water solubility and a high metabolic rate, both of which impede its use in clinical applications (Zhang M. et al., 2021).

Codrugs, also known as mutual prodrugs, are composed of two or more pharmacologically active agents to improve the therapeutic efficiency or decrease adverse effects. Codrug, as a classical drug design approach, has been widely used to improve biopharmaceuticals in various application areas (Das et al., 2010; Aljuffali et al., 2016) such as the antiviral agent remdesivir. Remdesivir, a nucleotide analog prodrug, has been approved for treating COVID-19 (Lamb, 2020; Lee et al., 2022). Thus, the synthesis of a novel antiplatelet agent featuring combined ozagrel and paeonol activity may improve the bioactivity.

In our previous study, the prodrug POC (C_22_H_20_N_2_O_4_, a codrug of ozagrel and paeonal) was synthesized, but it was found to be unstable. Consequently, the purpose of this research was to synthesize a series of novel codrugs of ozagrel and paeonol and evaluate their antiplatelet activity. The optimal compound PNC3 (8b) was chosen for further investigation, which included assessing its pharmacokinetic properties and its protective effect on OGD/R PC12 cells *in vitro*. Molecular docking was also used to predict the binding affinity of the most effective synthesized prodrugs with ADP and TXA2.

## 2 Materials and methods

### 2.1 Chemicals and instrumentation

All regents, solvents, and starting compounds are commercially available. The progress of the reactions was monitored via thin-layer chromatography (TLC) on silica gel GF_254_ plates (Haiwan Chemical, Qingdao, China) using a petroleum/ethyl acetate eluent. Column chromatography was used for the separation of intermediate products. Bruker AVANCE III 400 instruments were used to measure ^1^H and ^13^C NMR spectra with chemical shifts given in ppm (δ) (Bruker, Karlsruhe, Germany). The ^1^H and ^13^C NMR spectra were acquired in DMSO-d_6_ or CDCl_3_ with operating frequencies of 400 or 100 MHz, respectively. All ESI-MS spectra were obtained using a Thermo Fisher Scientific combined ion trap and Orbitrap high-resolution mass spectrometer furnished with an LTQ Orbitrap XL Mass Spectrometer (Thermo Fisher Scientific, TX, United States).

#### 2.1.1 Synthesis of the intermediate 3-(4-((1H-imidazol-1-yl)methyl)phenyl)acryloyl chloride (2)

Ozagrel (0.165 g, 0.00073 mol) was added to a DMF solution (0.05 mL). SOCl_2_ (0.5 mL) was then added dropwise to the mixture and stirred until it was completely dissolved at room temperature. The reaction system was refluxed for 5 h at 80°C in an oil bath. The resulting reaction system was concentrated using a rotary evaporator under reduced pressure after monitoring by TLC. The residue was purified via silica gel column chromatography (ethyl acetate) to yield intermediate **2**.

#### 2.1.2 Synthesis of 3-(4-((1H-imidazol-1-yl)methyl)phenyl)-N-(2-acetyl-5-metho-xyphenyl)propyl acrylamide (3, PNC_1_)

The paeonol derivative (2-amino-4-methoxyacetophenone, 0.1 g, 0.00060 mol) was dissolved in CH_2_Cl_2_ and mixed with TEA (0.3 g, 0.0030 mol). The mixture was slowly added to intermediate **2** under the ice bath. The reaction mixture was then sealed with a nitrogen balloon for 3 h at room temperature. The reaction was monitored by TLC. Target compound **3** was extracted repeatedly with saturated NaHCO_3_ and CH_2_Cl_2_. The organic layer was dehydrated with anhydrous Na_2_SO_4_ for 2 h and then concentrated. The residue was purified by column chromatography utilizing ethyl acetate/petroleum ether in a ratio of 4:1 (0.26 g, 10.92%).

#### 2.1.3 Synthesis of the intermediate 3-(4(1H-imidazol-1-yl)methyl)phenyl)-1-(piperazin-1-yl)prop-2-en-1-one (5)

Ozagrel (3 g, 0.013 mol), EDCl (4.16 g, 0.022 mol), and HOBT (2.94 g, 0.022 mol) were dissolved in CH_2_Cl_2_. After stirring for 2 h, the mixture was supplemented with TEA (6.58 g, 0.065 mol) and N-BOC-3-chloropropylamine (N-BOC, 4.04 g, 0.021 mol) and stirred for another hour. The reaction was monitored by TLC. After saturating the sodium bicarbonate water, it was added to the mixture, which was then extracted with CH_2_Cl_2_, dried with anhydrous Na_2_SO_4_, and concentrated under reduced pressure. The mixture was separated by column chromatography (ethyl acetate/petroleum ether = 3:1) to obtain compound **4**. The BOC group was removed with TFA (5 mL) in 20 mL CH_2_Cl_2_, and the solvent was evaporated under reduced pressure to obtain a yellow transparent solid compound **5**.

#### 2.1.4 General procedure for intermediates (7a–c)

The substituted chloro-bromo-alkyl (0.018 mol), anhydrous K_2_CO_3_ (2.49 g, 0.018 mol), and paeonol (3 g, 0.018 mol) were added to acetonitrile (10 mL). The reaction mixture was then stirred at 45°C for 4 h with N_2_ protection. The progress of the reaction was tracked by TLC. The product was extracted and purified in the same way as done previously.

#### 2.1.5 General procedure for target compounds (8a–c, PNC_2-4_)

Intermediate **5** (0.0033 mol), a 1.1-fold amount of intermediates **7a**–**c**, and anhydrous K_2_CO_3_ were added to a solution of DMF (5 mL), which was stirred at 45°C for 4 h with N_2_ protection. The reaction progress was monitored by TLC. Subsequently, the mixture was concentrated under reduced pressure, and the resulting residue was purified via column chromatography using ethyl acetate.

#### 2.1.6 Synthesis of intermediate 1-(2-(3-aminopropoxy)-4-methoxyphenyl)ethan-1-one (10)

Paeonol (0.206 g, 0.0012 mol) and a 1.1-fold amount of K_2_CO_3_ were added to DMF (2 mL). The mixture was agitated at 40°C for 4 h. The resulting white powder of compound **9** (0.00020 g, 58.9%) was extracted with CH_2_Cl_2_ and saturated with NaHCO_3_ after monitoring its progress on TLC. The mixture was then concentrated and purified by reduced pressure and column chromatography (ethyl acetate/petroleum ether = 1:4). Compound 9 was then treated with TFA (0.6 mL) in 2.4 mL of CH_2_Cl_2_ to remove the BOC group and obtain intermediate **10** (125 mg, 52%).

#### 2.1.7 Synthesis of the targeted compound 3-(4-((1H-imidazol-1-yl)methyl)phenyl)-N-(3-(2-acetyl-5-met-hoxyphenoxy)propyl)acrylamide (11, PNC_5_)

Ozagrel (0.141 g, 0.00085 mol), EDCL (0.118 g, 0.0006 mol), and HOBT (0.083 g, 0.0006 mol) were added to CH_2_Cl_2_ (2 mL) and stirred for 2 h. Then, intermediate **10** (0.125 g, 0.00056 mol) and a 3-fold amount of TEA (0.0025 mol) were added to the current reaction and stirred for an additional 1 h. The separated compound was filtered through column chromatography (ethyl acetate/petroleum ether/methanol = 4:8:1) after monitoring by TLC. This process yielded compound **11** (116 mg, 43.8%).

### 2.2 Antiplatelet aggregation activity

The antiplatelet aggregation activity of the target compounds was evaluated against ADP- and AA-induced platelet aggregation. In other words, different concentrations of the target compound solution and POC (Zhang J. et al., 2021) (10 μL) were added to the plasma at 37°C for 5 min. Then, platelets were stimulated with ADP (10 μM, 30 μL) or AA (1 mM, 30 μL). Variations in absorbance were monitored for 5 min in order to measure platelet aggregation (Daron et al., 2022). The platelet aggregation inhibition rate (AIR) was calculated according to the following formula: AIR = (rate of aggregation (control)−rate of aggregation (compounds))/rate of aggregation (control) × 100%

### 2.3 Pharmacokinetic analysis

Six rats were given compound **8b** (6.4 mg/kg) (Zhang J. et al., 2021) by intravenous injection (i.v.) and intragastric administration (i.g.), respectively. The animal experiment protocol was performed according to the guidelines for animal experiments and supervised by the Experimental Animal Ethics Committee of Anhui University of Chinese Medicine (AHUCM-rats-2019001). Then, approximately 1.0 mL of blood was taken from the orbital venous plexus of the rats and placed into an EP-containing heparin at predetermined time points of 3, 5, 10, 20, 30, 45, 60, 120, and 240 min after dosing. The blood sample was centrifuged for 15 min at 3,500 rpm to obtain plasma.

For plasma preparation, 180 μL of plasma was mixed with 20 μL of glibenclamide as an internal standard and 900 μL of acetonitrile. The mixture was then centrifuged at 12,000 rpm for 10 min. After centrifugation, 800 μL of the supernatant was dried using a vacuum freeze dryer. The dried sample was then reconstituted with 200 μL of acetonitrile for LC/MS analysis.

The analytical conditions for the plasma samples are as follows: a Hypersil GOLD C8 HPLC Column (2.1 mm × 100 mm, 1.9μm; Thermo Fisher Scientific, United States) was used for chromatographic separation. The column temperature was maintained at 30°C, and the sample chamber temperature was maintained at 4°C. A gradient elution with a flow rate of 0.2 mL/min (phase A: 0.1% formic acid, phase B: methanol) was carried out from 45% B to 80% within 2.5 min, then from 85% to 90% within 1 min, and maintained at 90% for 1 min; subsequently, it was decreased from 90% to 45% in 30 s and maintained for 1 min.

Multiple-reaction monitoring (MRM) was quoted as the acquisition mode of mass spectrometry in a positive ionization mode. The mass spectrometry detector was equipped with an electrospray ionization (ESI) source. The optimal MS parameters were as follows: the ion source temperature was set at 400°C, the vaporization temperature was set at 500°C, the electrospray voltage was set to 5500 V, and the capillary voltage was set to 3600 V. The ion pair of **8b** is m/z 517.0 and 281.4, and the declustering voltage is 120 eV; the collision energy is 40 eV. Similarly, the ion pair of glibenclamide is m/z 494.1 and 169.0, the declustering voltage is 65 eV, and the collision energy is 50 eV.

### 2.4 Cell culture and anti-oxygen-glucose deprivation cell activity

For the cell culture conditions, rat pheochromocytoma PC12 cells (ATCC, Manassas, VA, United States) were cultured in RPMI 1640 culture medium with 1% penicillin and streptomycin and 10% fetal bovine serum (FBS) (Gibco, Waltham, MA, United States) at 37°C with 5% CO_2_.

Cell viability was assessed using a CCK-8 assay. The PC12 cells were seeded in a 96-well plate at a density of 1 × 10^5^ cells per well. After incubation for 24 h, the cells were treated with various concentrations of the compound. After 24 h of re-incubation, the cell viability was evaluated using a 10% CCK-8 solution. The absorbance value was determined at 450 nm.

For oxygen-glucose deprivation of PC12 cell activity, the procedure was conducted as follows (Wei et al., 2022): the cells were cultured in a 96-well plate for 24 h. Then, the culture medium was replaced with oxygen–glucose-free RPMI 1640 and placed in an incubator with 5% CO_2_ and 95% N_2_ for 2 h. Once the oxygen-free process was completed, the culture medium was changed back to RPMI 1640 and maintained at 37°C with 5% CO_2_ for an additional 24 h. Cell viability was then assessed using the CCK-8 assay.

### 2.5 Molecular docking

Molecular docking was conducted using the CDOCKER protocol and the receptor–ligand interaction protocol of Discover Studio Client software. Different scorings of the CDOCKER protocol were implemented for the ligands, including CDOCKER_ENERGY (internal and receptor–ligand strain energy) and CDOCKER_INTERACTION_ENERGY (interaction energy). Molecular docking was based on the crystal structures of P2Y12 (PDB ID: 4PXZ) and TXA2 (PDB ID: 6IIU), where the proteins bind to ozagrel, paeonal, and PNC_3_ as ligands. The X-ray crystal structures of P2Y12 and TXA2 were obtained from the RCSB Protein Data Bank (PDB) (https://www.rcsb.org/). The poses were sorted according to CDOCKER_ENERGY, and the poses with the highest CDOCKER_ENERGY values were analyzed and mapped.

## 3 Results

### 3.1 Chemistry

**FIGURE 1:**
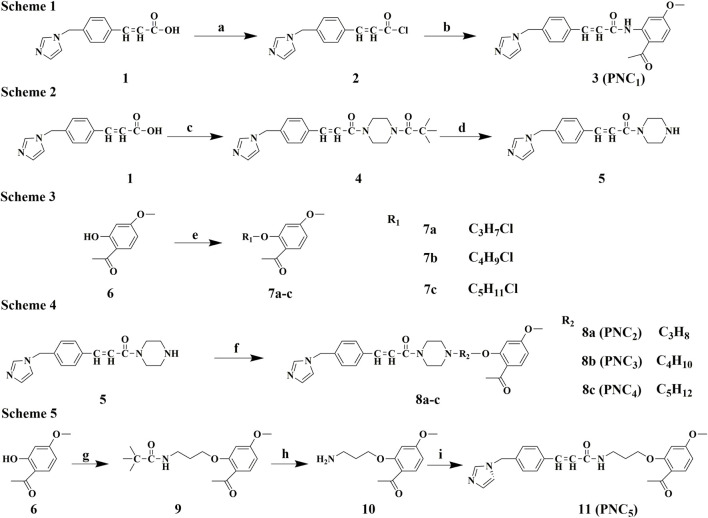
Synthesis route of PNC_1–5_. **Scheme 1**. Synthesis of target compound 3 (PNC_1_). Conditions: (a) DMF, CH_2_Cl_2_, SOCl_2_, 80°C, reflux, and 5 h; (b) paeonol, DCM, rt, and 3 h. **Scheme 2**. Synthesis of intermediate 5. Conditions: (c) N-t-Butyloxy carbonyl, 1-hydro-xybenzotriazole, EDCl, TEA, DCM, rt, and 1 h; (d) TFA, DCM, rt, and 5 h. **Scheme 3**. Synthesis of intermediates 7a–c. Conditions: (e) C_x_H_y_BrCl (x = 3, 4, and 5; y = 6, 8, and 10), DMF, K_2_CO_3_, 45°C, and 4 h. **Scheme 4**. Synthesis of target compounds 8a–c (PNC_2-4_). Conditions: (f) 7a–c, DMF, K_2_CO_3_, 45°C, and 4 h. **Scheme 5**. Synthesis of target compound 11 (PNC_5_). Conditions: (g)1-(4-(3-chloropro-pyl)piperazin-1-yl)-2,2-dimethylpropan-1-one, DMF, K_2_CO_3_, 45°C, and 4 h; (h) TFA, DCM, rt, and 5 h; (i) ozagrel, 1-hydroxybenzotriazole, EDCl, TEA, DCM, rt, and 1 h.

The synthetic routes of intermediates **2**, **5**, and **7a–c** are outlined in schemes 1, 2, and 3 (Figure 1), respectively. According to a known procedure, intermediate **2** was generated using ozagrel to react with thionyl chloride (SOCl_2_) and *N*, *N*-dimethylformamide (DMF) (Zhang J. et al., 2021). Intermediate **5** with a piperazine ring was obtained by a nucleophilic substitution reaction in the presence of triethylamine (TEA) and CH_2_Cl_2_. Intermediates **7a–c** were produced by allowing paeonol to react with chloro-bromo-alkyl compounds of different carbon chain lengths.

The synthetic routes for target compounds **3**, **8a–c**, and **11** are outlined in schemes 1, 4, and 5 (Figure 1). In the presence of potassium carbonate in DMF, treatment of intermediate **5** with the corresponding intermediates **7a–c** at 45°C for 4 h yields compounds **8a–c**. Intermediates **2** and **10** react with the corresponding CH_2_Cl_2_ in the presence of *N*-(3-dimethylaminopropyl)-*N′*-ethylcarbodiimide hydrochloride (EDCl), 1-hydroxybenzotriazole (HOBT), and TEA at room temperature for 2 h, resulting in the synthesis of target compounds **3** and **11**.

#### 3.1.1 3-(4-((1H-imidazol-1-yl)methyl)phenyl)-N-(2-acetyl-5-metho-xyphenyl)propyl acrylamide (3, PNC_1_) yield: 10.92%

^1^H-NMR (400 MHz, DMSO-d6): δ 12.49 (s, 1H), 8.63 (d, 2.4 Hz, 1H), 7.89 (d, 8.8 Hz, 1H), 7.76 (d, 15.6 Hz, 1H), 7.68 (s, 1H), 7.63 (s, 1H), 7.61 (s, 1H), 7.24 (s, 1H), 7.22 (s, 1H), 7.17 (s, 1H), 6.96 (s, 1H), 6.69 (s, 1H), 5.20 (s, 2H), 3.96 (s, 3H), and 2.68 (s, 3H). ^13^C-NMR (100 MHz, CDCl3): δ 201.36, 164.95, 164.90, 144.00, 141.22, 137.45, 134.95, 133.73, 130.04, 129.73, 129.71, 128.73, 127.92, 123.00, 115.27, 110.07, 104.07, 55.66, 50.81, and 29.71. LC-MS (ESI), with m/z calculated for C_22_H_21_N_3_O_3_ as 376.1661, found an observed value of [M + H]^+^ 376.1650.

#### 3.1.2 3-(4-((1H-imidazol-1-yl)methyl)phenyl)-1-(4-(3-(2-acetyl-5-metho-xyphenyl)oxy) propyl)piperazin-1-yl)prop-2-en-1-one (8a, PNC_2_) yield: 34.6%

^1^H-NMR (400 MHz, DMSO-d6): δ 7.78 (t, 1.2 Hz, 1H), 7.71 (s, 1H), 7.69 (s, 1H), 7.67 (d, 8.8 Hz, 1H), 7.46 (d, 15.6 Hz, 1H), 7.28 (s, 1H), 7.26 (s, 1H), 7.25 (d, 15.2 Hz, 1H), 7.20 (t, 1.2 Hz, 1H), 6.92 (t, 1.2 Hz, 1H), 6.63 (d, 2.4 Hz, 1H), 6.60 (dd, 8.4, 2.0 Hz, 1H), 5.22 (s, 2H), 4.16 (t, 6.0 Hz, 2H), 3.83 (s, 3H), 3.75–3.65 (m, 2H), 3.63–3.53 (m, 2H), 2.56–2.52 (m, 2H), 2.51–2.50 (m, 3H), 2.47–2.38 (m, 4H), and 2.04–1.94 (m, 2H). ^13^C-NMR (100 MHz, CDCl3): δ 197.66, 165.17, 164.51, 160.35, 141.88, 137.55, 137.47, 135.38, 132.77, 129.87, 128.34, 127.70, 121.22, 119.30, 117.75, 105.02, 99.13, 66.55, 55.58, 55.04, 53.68, 52.82, 50.50, 45.75, 42.09, 32.02, and 26.47. LC-MS (ESI), with m/z calculated for C_29_H_34_N_4_O_4_ as 503.2658, found an observed value of [M + H]^+^ 503.2639.

#### 3.1.3 3-(4-((1H-imidazol-1-yl)methyl)phenyl)-1-(4-(4-(2-acetyl-5-metho-xyphenyl) oxy)butyl)piperazin-1-yl)prop-2-en-1-one (8b, PNC_3_) yield: 44.6%

^1^H-NMR (400 MHz, DMSO-d6): δ 7.76 (t, 1.2 Hz, 1H), 7.71 (s, 1H), 7.69 (s, 1H), 7.67 (d, 8.4 Hz, 1H), 7.46 (d, 15.2 Hz, 1H), 7.28 (s, 1H), 7.26 (s, 1H), 7.24 (d, 15.2 Hz, 1H),7.19 (t, 1.2 Hz, 1H), 6.91 (t, 1.2 Hz, 1H), 6.64 (d, 2.4 Hz, 1H), 6.59 (dd, 8.8, 2.4 Hz, 1H), 5.21 (s, 2H), 4.13 (t, 6.0 Hz, 2H), 3.83 (s, 3H), 3.73–3.63 (m, 2H), 3.61–3.50 (m, 2H), 2.50–2.48 (m, 3H), 2.43–2.33 (m, 6H), 1.90–1.78 (m, 2H), and 1.70–1.58 (m, 2H). ^13^C-NMR (100 MHz, CDCl3): δ 197.70, 165.14, 164.48, 160.43, 141.77, 137.57, 137.48, 135.39, 132.72, 130.03, 128.31, 127.66, 121.25, 119.27, 117.81, 104.96, 99.01, 68.26, 57.90, 55.55, 53.50, 52.86, 50.45, 45.82, 42.17, 32.07, 27.08, and 23.47. LC-MS (ESI), with m/z calculated for C_30_H_36_N_4_O_4_ as 517.2815, found an observed value of [M + H]^+^ 517.2799.

3-(4-((1H-imidazol-1-yl)methyl)phenyl)-1-(4-(5-(2-acetyl-5-metho-xyphenyl) oxy) pentyl)piperazin-1-yl)prop-2-en-1-one (8c, PNC_4_) yield: 22.8%

^1^H-NMR (400 MHz, DMSO-d6): δ 6.77 (t, 1.2 Hz, 1H), 6.72 (s, 1H), 6.70 (s, 1H), 6.67 (d, 8.4 Hz, 1H), 6.47 (d, 15.2 Hz, 1H), 6.29 (s, 1H), 6.27 (s, 1H), 6.25 (d, 15.2 Hz, 1H), 6.20 (t, 1.2 Hz, 1H), 5.92 (t, 1.2 Hz, 1H), 5.65 (d, 2.4 Hz, 1H), 5.60 (dd, 8.8, 2.4 Hz, 1H), 4.22 (s, 2H), 3.12 (t, 6.0 Hz, 2H), 2.84 (s, 3H), 2.73–2.64 (m, 2H), 2.60–2.50 (m, 2H), 1.43–1.29 (m, 6H), 0.88–0.76 (m, 2H), and 0.60–0.44 (m, 4H). ^13^C-NMR (100 MHz, CDCl3): δ 197.76, 165.14, 164.49, 160.51, 141.75, 137.55, 137.48, 135.40, 132.70, 130.00, 128.31, 127.66, 121.22, 119.28, 117.84, 104.97, 98.92, 68.31, 58.22, 55.54, 53.59, 52.83, 50.71, 50.46, 45.84, 42.53, 32.10, 29.05, 26.50, and 24.17. LC-MS (ESI), with m/z calculated for C_31_H_38_N_4_O_4_ as 531.2971, found an observed value of [M + H]^+^531.2970.

#### 3.1.4 3-(4-((1H-imidazol-1-yl)methyl)phenyl)-N-(3-(2-acetyl-5-metho-xyphenoxy) propyl)acrylamide (11, PNC_5_) yield: 72.6%

^1^H-NMR (400 MHz, DMSO-d6): δ 8.27 (t, 5.6 Hz, 1H), 7.76 (t, 1.2 Hz, 1H), 7.68 (d, 8.4 Hz, 1H), 7.55 (s, 1H), 7.53 (s, 1H), 7.40 (d, 16.0 Hz, 1H), 7.27 (s, 1H), 7.25 (s, 1H), 7.19 (t, 1.2 Hz, 1H), 6.92 (t, 1.2 Hz, 1H), 6.63 (d, 2.4 Hz, 2H), 6.61 (d, 2.4 Hz, 2H), 6.59 (d, 2.4 Hz, 1H), 5.21 (s, 2H), 4.14 (t, 6.0 Hz, 2H), 3.82 (s, 3H), 3.41–3.38 (m, 2H), 2.53 (s, 3H), and 2.00 (q, 6.4 Hz, 2H). ^13^C-NMR (100 MHz, CDCl3) δ 197.32, 166.33, 164.71, 160.16, 138.80, 137.47, 136.99, 135.77, 133.90, 129.88, 128.29, 127.55, 122.77, 119.79, 119.35, 104.71, 99.16, 68.58, 55.61, 50.70, 50.52, 38.54, 30.12, 29.70, and 28.63. LC-MS (ESI), with m/z calculated for C_25_H_27_N_3_O_4_ as 434.2080, found an observed value of [M + H]^+^ 434.2064.

The target compounds were synthesized using the described method, with ^1^H-NMR, ^13^C-NMR, and m/z data available in the Supporting Information.

### 3.2 Antiplatelet aggregation activity

**FIGURE 2:**
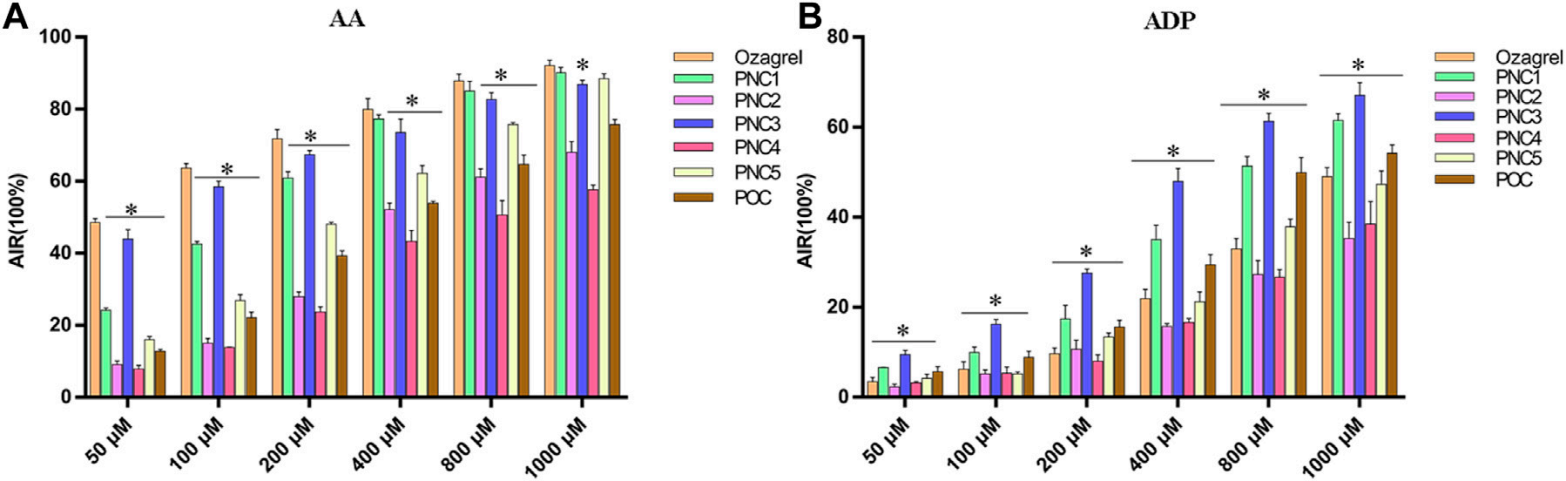
Antiplatelet aggregation activity of the target compounds in different concentrations. **(A)** Target compound on AA-induced platelet aggregation. **(B)** Target compound on ADP-induced platelet aggregation. *p < 0.05 vs. ozagrel group.

To assess the effect of the target compounds on antiplatelet aggregation activity, AA- and ADP-induced platelet aggregation were evaluated. The synthesized target compounds (PNC_1-5_) and POC displayed a remarkable inhibitory effect on platelet aggregation, with IC_50_ values ranging from 485 to 1,000 μM and 52.46–692.40 μM for ADP- and AA-induced platelet aggregation, respectively (Table 1). Notably, all the target compounds were more effective in inhibiting AA-induced platelet aggregation than ADP-induced aggregation. Among the target compounds, PNC_1_ and PNC_3_ demonstrated the most effective antiplatelet aggregation activity, displaying a dose-dependent response between 50 and 1,000 μM (Figure 2). Notably, their antiplatelet aggregation activity was superior to that of POC. PNC_3_ was the most effective of all the target compounds, exhibiting the strongest antiplatelet activity in response to ADP- and AA-induced platelet aggregation. Consequently, PNC3 was the main focus of subsequent studies.

**TABLE 1:** Antiplatelet aggregation activity of the target compounds (IC_50_, μM).

Compound	IC_50_ (μM)	
	ADP (10 μM)	AA (1 μM)
Ozagrel	>1,000	52.46 ± 3.29
PNC_1_	710 ± 43.71^**^	135.4 ± 10.86^#^
PNC_2_	>1,000	462 ± 28.73
PNC_3_	485 ± 31.07^**#^	74.7 ± 4.86^##^
PNC_4_	>1,000	692.4 ± 45.73
PNC_5_	>1,000	233.8 ± 16.55
POC	842 ± 53.29	349.2 ± 15.97

*/# represents *p* < 0.05; **/## represents *p* < 0.01; * represents comparison with the ozagrel group; and # represents comparison with the POC group. Data are expressed as the mean ± SD, *n* = 5.

### 3.3 Pharmacokinetic study of PNC_3_

**FIGURE 3:**
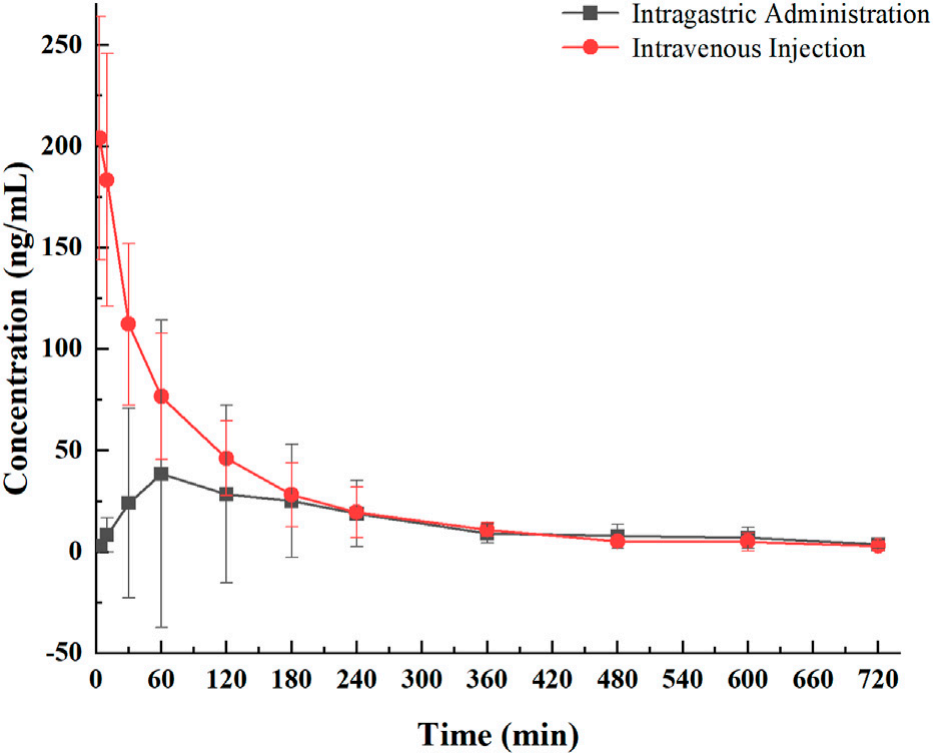
Pharmacological curve of compound PNC_3_ after a single i.g. or i.v. administration to rats.

To evaluate the release profile of PNC_3_ *in vivo*, pharmacokinetic (PK) studies were conducted. These studies revealed that PNC_3_ was immediately detected in the blood when administered intravenously (i.v.), with a peak concentration visible within 6 h. When administered intragastrically (i.g.), the peak concentration of PNC_3_ in the blood was observed at 1 h and remained detectable for up to 8 h (Figure 3). Despite PNC_3_ having a lower blood concentration, the AUC was still achieved at 33.3% when administered by i.g (Table 2). It was shown that PNC3 is capable of being absorbed into the blood through the intestine. Notably, the t_1/2_ and MRT of PNC_3_ administered by i.g. were 2.33 and 2.11 times longer than those of the drug administered by i.v., respectively, indicating that PNC_3_ has the potential to be developed as an oral formulation.

**TABLE 2:** Key pharmacokinetic parameters of compound PNC_3_ after a single (6.4 mg/kg) i.g. or i.v. administration to rats.

Parameter	i.g.	i.v.
AUC(0-t)/(μg/L*min)	6148.338 ± 2222.737	18,552.773 ± 7438.264
AUC(0-∞)/(μg/L*min)	8346.219 ± 3,719.156	19,250.669 ± 7673.11
t_1/2_/(min)	290.961 ± 104.671	124.945 ± 17.725
MRT (0-t)/(min)	316.761 ± 46.915	149.789 ± 62.038

t_1/2_, half-life; MRT, mean resident time. Data are reported as the mean ± SD, *n* = 6.

### 3.4 Assessment of the protective effect on OGD/R cells

**FIGURE 4:**
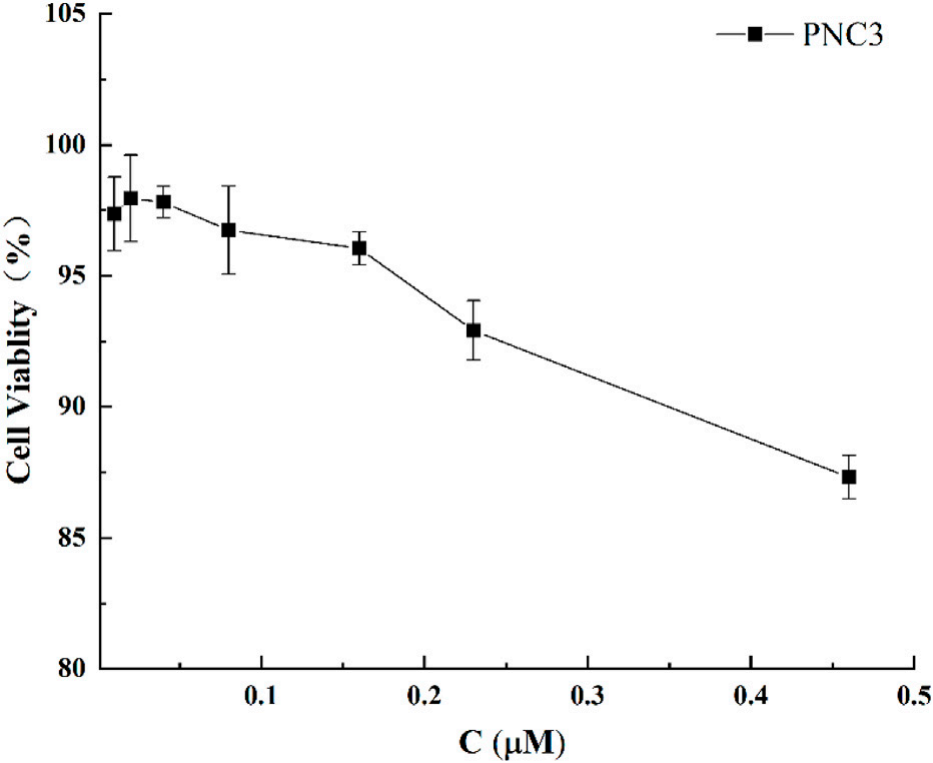
Effect of PNC_3_ on the viability of PC12 cells with different concentrations (0.01–0.46 μM).

To exclude the effect of PNC_3_ on PC12 cells, the viability of PC12 cells treated with different PNC_3_ concentrations (0.01–0.46 μM) for 24 h was measured using CCK-8 assay. As shown in Figure 4, a PNC_3_ concentration of 0–0.16 μM had no impact on cell viability, with a cell survival rate of 97.52% at 0.08 μM. When the concentration exceeded 0.16 μM, the cell activity began to decrease, with a cell survival rate of 94.03%. Therefore, 0.08 μM PNC_3_ treatment was selected to establish the high concentration for OGD/R.

To evaluate the neuroprotective potential of PNC_3_, PC12 cells were exposed to oxygen-glucose deprivation. The medium in this process all contained PNC_3_ (0.02, 0.04, and 0.08 μM) in the PNC_3_ group. The cell viability at 0.02 μM PNC_3_ was not superior to that at 0.04 and 0.08 μM (Table 3), suggesting that PNC_3_ exhibits a neuroprotective effect with a certain level of dose-dependence.

**TABLE 3:** Effect of PNC_3_ on the viability of OGD/R PC12 cells.

Cell type	Compound	Group (μM)	Cell viability (%)
PC12	PNC3	Control	100 ± 2.06
		OGD/R	49.59 ± 3.14^###^
		0.02	50.54 ± 2.95
		0.04	54.22 ± 2.68^*^
		0.08	62.26 ± 3.14^***^

^#^ represents comparison with the control group; * represents comparison with the OGD/R group; */^#^ represents *p* < 0.05; and ***/^###^ represents *p* < 0.01. Data are expressed as the mean ± SD, *n* = =5.

### 3.5 Molecular docking

To investigate the potential targets of ozagrel, paeonol, and PNC_3_, molecular docking analysis was conducted. The study focused on two crucial proteins involved in platelet aggregation: P2Y12 and TXA2.

The CDOCKER_ENERGY (23.5716 and 17.2549 kcal/mol) and CDOCKER_INTERACTION_ENERGY (−47.581 and −40.7488 kcal/mol) of PNC_3_ with P2Y12 and TXA2 were superior to those of ozagrel and paeonol (Table 4). These results indicated that PNC_3_ has the potential to inhibit platelet aggregation.

**TABLE 4:** Energy value of PNC3, ozagrel, and paeonol in molecular docking.

Ligand	Protein	CDOCKER_ENERGY (kcal/mol)	CDOCKER_INTERACTION_ENERGY (kcal/mol)
PNC3	4PXZ	23.5716	−47.581
Ozagrel		23.3469	−25.103
Paeonol		15.6299	−21.1253
PNC3	6IIU	17.2549	−40.7488
Ozagrel		16.3212	−17.1106
Paeonol		8.42412	−14.0715

**FIGURE 5:**
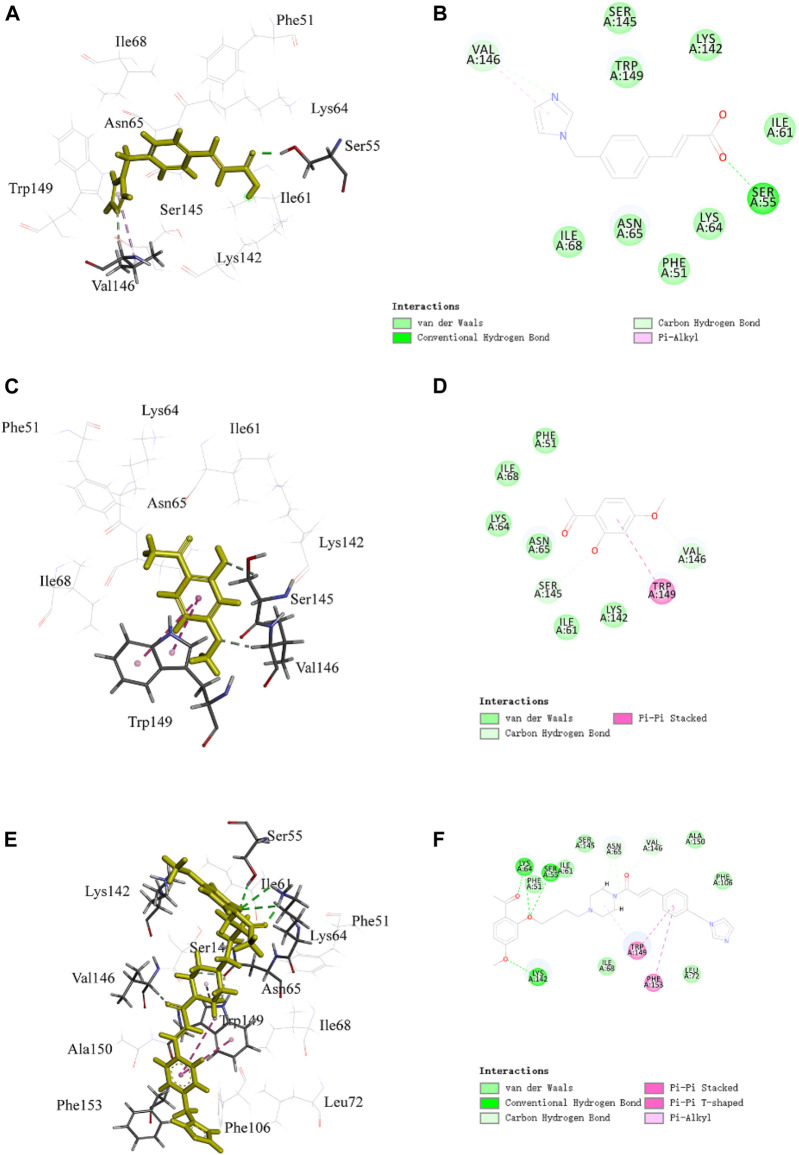
Representative image of the docking of PNC_3_, ozagrel and paeonol; the binding site of P2Y12: **(A,C,E)** 3D image and **(B,D,F)** 2D image, respectively.

**FIGURE 6:**
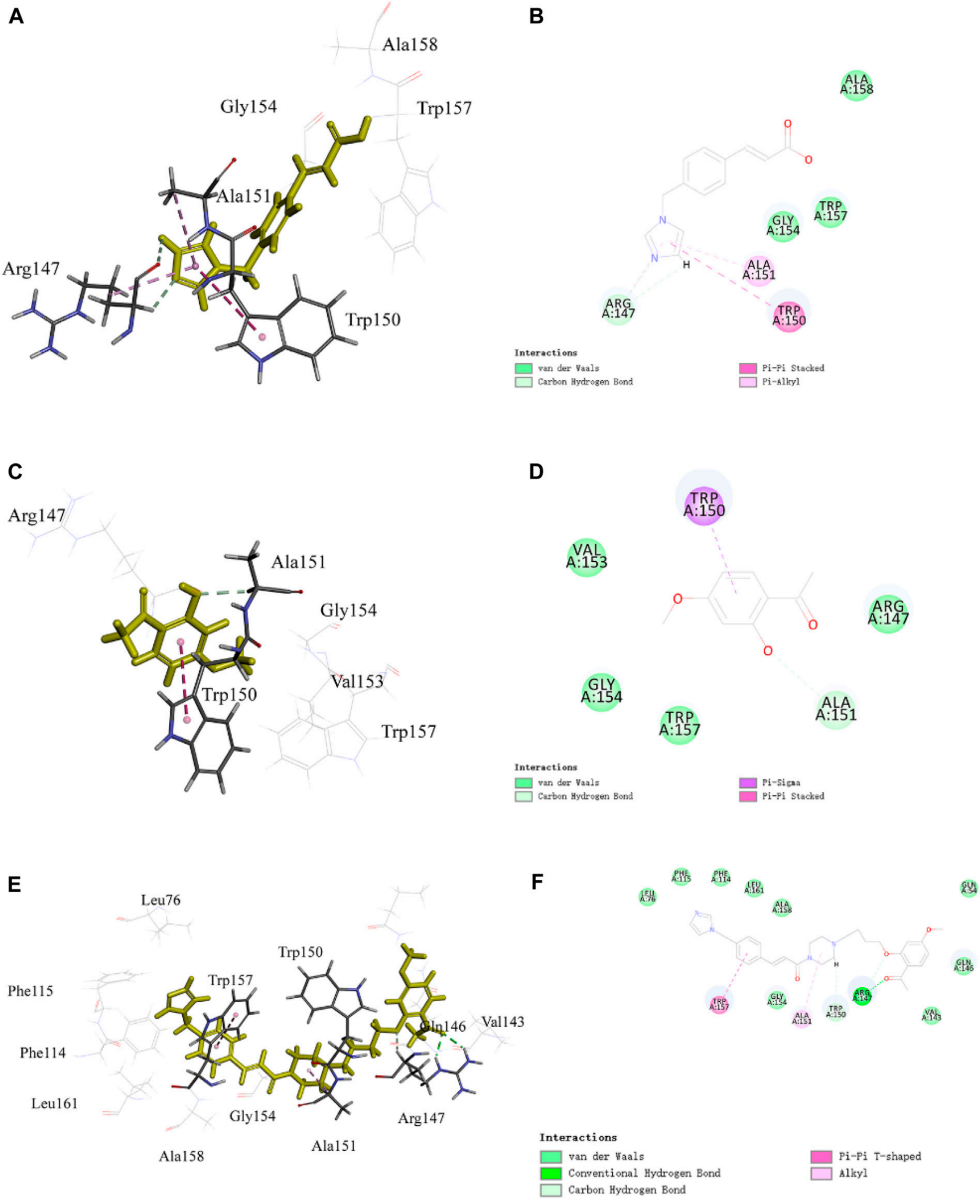
Representative image of the docking of PNC_3_, ozagrel, and paeonol; the binding site of TXA2: **(A,C,E)** 3D image and **(B,D,F)** 2D image, respectively.

**FIGURE 7:**
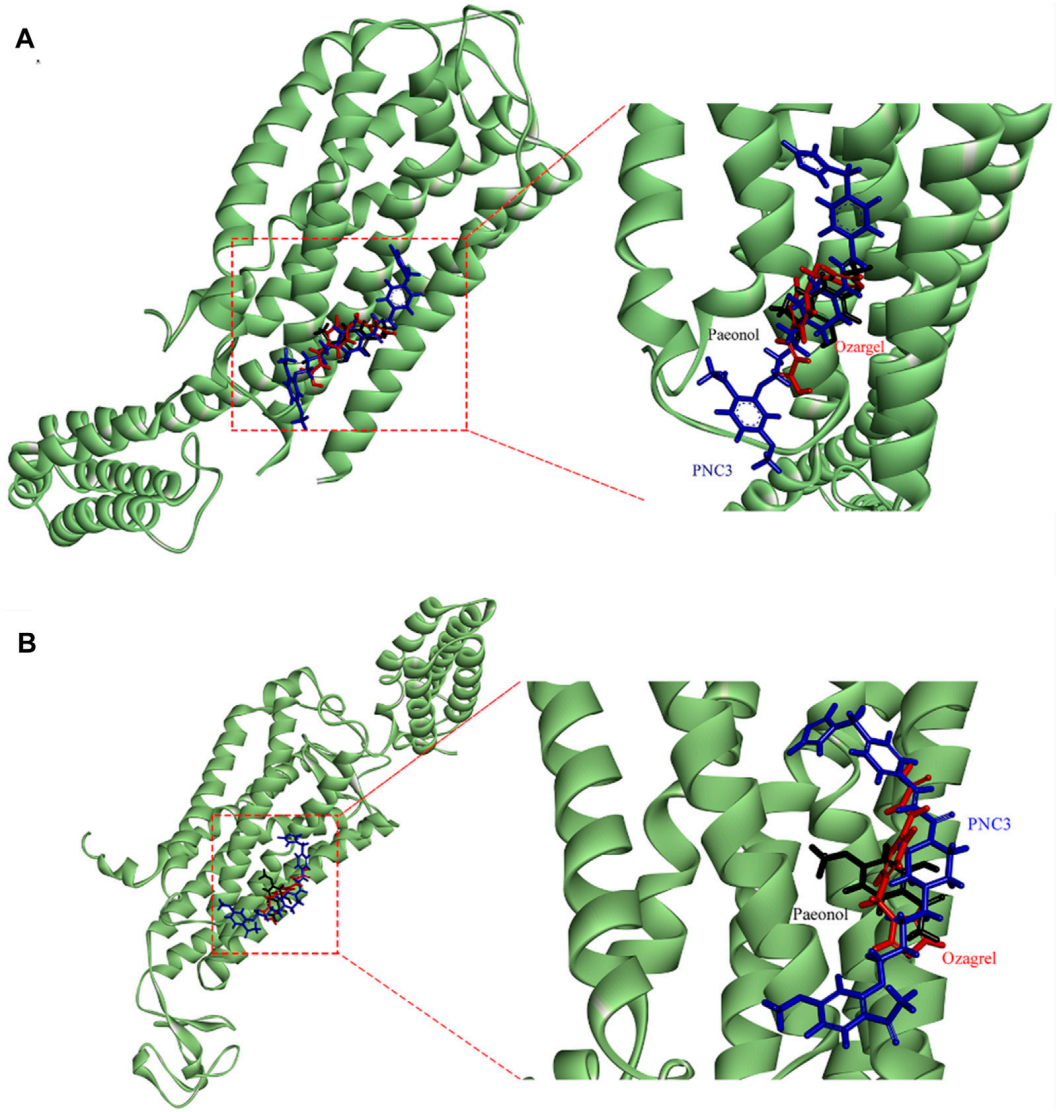
Stacking map of PNC3, ozagrel, and paeonol. **(A)** Binding with P2Y12. **(B)** Binding with TXA2.

For PNC_3_, strong inhibition of platelet aggregation was observed, which encouraged us to explore their potential binding modes with P2Y12 and TXA2 using a molecular docking approach. The binding modes of PNC_3_, ozagrel, and paeonol with P2Y12 and TXA2 are shown in Figures 5–7.

The benzene ring group of PNC_3_ effectively engages Trp149 through π–π stacking and carbon–hydrogen bonding, while the benzene ring group of paeonol forms π–π stacking with Trp149. Additionally, the carbonyl group of PNC_3_ forms a π–alkyl bond with Val146, while the pyrazole group of ozagrel forms a π–alkyl bond with Val146, and the oxygen atom of paeonol forms a carbon–hydrogen bond with Val146. Furthermore, the oxygen atom of PNC_3_ forms a conventional hydrogen bond with Ser55, while the pyrazole group of ozagrel forms a conventional hydrogen bond with Ser55. These results suggest that PNC_3_, ozagrel, and paeonol have similar docking sites, indicating that inhibiting the P2Y12 protein with PNC_3_ is completely feasible.

The pyrazine group of PNC_3_ effectively engages Ala151 and Trp150 through alkyl and carbon–hydrogen bonds, while the pyrazole group of ozagrel forms a π–alkyl bond with Ala151 and π–π stacks with Trp150. The benzene ring group of paeonol forms a π–sigma bond with Trp150, and the oxygen atom of paeonol forms a carbon–hydrogen bond with Ala151. Furthermore, the oxygen atom and carbonyl group of PNC_3_ effectively engage Arg147 through van der Waals and conventional hydrogen bonds, while the pyrazole group of paeonol forms a π–alkyl bond, a van der Waals bond, and a carbon–hydrogen bond with Arg147. These results show that PNC_3_, ozagrel, and paeonol have similar docking sites, suggesting that the TXA2 protein can be inhibited with PNC_3_.

## 4 Discussion

It is well-known that ozagrel and paeonol have beneficial pharmacological effects in stroke and antiplatelet aggregation. In our previous study, we synthesized POC using the combination principle, yet it was unstable and prone to decomposition. Additionally, ozagrel and paeonol are characterized by low oral bioavailability, which may be due to their poor water solubility (Mio et al., 2013; Zong et al., 2017). Drugs with good bioavailability need a certain lipid-water partition coefficient (Peng et al., 2008). Amino groups are frequently utilized in drug design due to their ability to improve water solubility and form hydrogen bonds that can interact with biological targets to maximize therapeutic efficacy (Connell et al., 2014; Nosov et al., 2018). In the synthesized PNC_1_, the amino group on the benzene ring is difficult to react with ozagrel amide because of its reduced activity. Therefore, ozagrel was chlorinated first to enhance the activity of the acyl chloride. In addition, the N-methylpiperazine ring, which plays a critical role in inhibiting platelet aggregation, was also introduced in the synthesized intermediate prodrugs: PNC_2_–PNC_4_. Different lengths of carbon chains were designed to explore their effects. Hydrogen undergoes nucleophilic substitution with chlorine or bromine in the synthesized PNC_2_–PNC_4_. Furthermore, the carboxylic acid group (-COOH) in ozagrel and the amino group in paeonol were masked by amide groups (-CONH) in the synthesized PNC_5_. Notably, N_2_ was used as a protective agent in the reaction to prevent oxidation and increase the yield of the synthesized PNC.

Thromboembolism is a classic feature of stroke. Thus, the antiplatelet aggregation activity of synthesized codrugs was investigated using AA- and ADP-induced platelet aggregation. The N-methylpiperazine ring and carbon chains in the synthesized codrugs are accountable for the variation in pharmacological activity between ozagrel and its prodrugs. The prodrug of PNC_3_ exhibited the most potent antiplatelet aggregation activity among the synthesized prodrugs, which can be attributed to the presence of paeonal, in addition to the N-methylpiperazine ring and carbon chains, when compared to other prodrugs such as PNC_1_ and PNC_5_. The prodrugs (PNC_3_) showed higher antiplatelet aggregation activity than the prodrugs PNC_2_ and PNC_4_. The former possesses proper carbon chains, while the latter possesses short or long carbon chains. This manifested antiplatelet activity may be due to the action on the carbon chains, resulting in an increased binding capacity of TXA_2_ and ADP receptors. Interestingly, the −CONR group of PNC_1_ by efficient hydrogen bonding has been proved to be useful in antiplatelet activity when compared with the previous prodrug POC. Based on our results, it is proposed that the amino, N-methylpiperazine ring, and carbon chains are more effective in antiplatelet activity than the ester group.

The next investigation focused on the pharmacokinetics, biological activity, and interactions with the target protein of PNC_3_, which is the most effective substance with antiplatelet aggregation activity in the synthesis of the target compounds. The pharmacokinetics of PNC_3_ was investigated to assess the potential of oral formulations, focusing on synthesized prodrugs with optimal activity. The ratio of AUC_(0-t)_ for i. v. administration compared to i. g. administration, accounting for 33.1%, elucidated that PNC_3_ can be absorbed into the blood through the intestine. In addition, i. g. MRT_(0-∞)_ was 2.11 times longer than that of i. v. administration, indicating that PNC_3_ may have the potential to be developed into an oral formulation. The improved AUC_(0-t)_ and MRT_(0-∞)_ may be attributed to the N-methylpiperazine ring and the appropriate length of carbon chains. To explore the possibility of PNC_3_ being developed as an oral formulation, an initial assessment of its protective effect on OGD/R PC12 cells was conducted. The PC12 cells, which exhibit neuronal properties, are commonly employed in stroke research (Chua and Lim, 2021). Stroke-induced cell damage occurs due to the deprivation of oxygen and energy supply to brain tissue (Zhang and Yang, 2021). Therefore, an *in vitro* model of oxygen-glucose deprivation/reperfusion (OGD/R) was established in this study to assess the protective effect of PNC_3_ on PC12 cells. This result suggests that PNC_3_ has a protective effect on OGD/R PC12 cells. Following this, molecular docking was conducted to validate the antiplatelet aggregation activity of PNC_3_. The CDOCKER score and the binding energy of P2Y12 and TXA2 with PNC_3_ were found to be satisfactory. P2Y12 is the primary receptor involved in ADP-induced platelet aggregation. The three p-alkyl bonds, conventional hydrogen bonds, and two amide pi-stacked and pi–pi T-shaped bonds formed between P2Y12 and PNC_3_ inhibit the function of P2Y12, consequently suppressing ADP-triggered platelet aggregation. Arachidonic acid (AA) within platelets undergoes conversion to TXA2 via intraplatelet thromboxane A2 synthase. The conventional hydrogen, pi–pi T-shaped, and alkyl bonds formed by TXA2 and PNC3 can be utilized to hinder TXA2 synthesis. Combining the outcomes of the PC12 cell assay and molecular docking assessment, it can be inferred that PNC_3_ binds P2Y12 and TXA2 efficiently, leading to the inhibition of ADP- and AA-induced platelet aggregation.

In this study, the codrug of ozagrel and paeonol (PNC_1_–PNC_5_) was synthesized with antiplatelet aggregation activity, protecting PC12 cells impaired by glucose deprivation and hypoxia and exhibiting good bioavailability. Nonetheless, the following aspects still require further exploration: 1) identification of potential by-products in the synthesis process that could potentially serve as effective drugs. 2) Utilizing molecular dynamics simulation to investigate the binding forces between target sites due to limited experimental facilities. 3) Formulation into liposomes holds promise for achieving targeted and sustained release. 4) Utilizing the structure–activity relationship (SAR) or quantitative structure–activity relationship (QSAR) to systematically understand the effect of various substituents on antiplatelet aggregation activity, expanding the range of applications for cardiovascular diseases.

In conclusion, the codrugs of ozagrel and paeonol with antiplatelet aggregation activity have been synthesized efficiently with good yields. The optimal prodrug of PNC_3_ among the PNC_S_ has good antiplatelet aggregation activity and effectively protects damaged PC12 cells from oxygen-glucose deprivation. The pharmacokinetic results indicate that PNC_3_ has the potential to be an oral formulation.

## Data Availability

The original contributions presented in the study are included in the article/Supplementary Material; further inquiries can be directed to the corresponding authors.
