# Supplementary material for: Design, synthesis, and evaluation of the novel ozagrel–paeonol codrug with antiplatelet aggregation activities as a potent anti-stroke therapeutic agent
Source: Front Pharmacol. 2024 Mar 19;15:1362857. doi: 10.3389/fphar.2024.1362857 (PMC10985144; doi:10.3389/fphar.2024.1362857)
Supplement: Supplementary file 1 [file Table1.DOCX]

**Design, synthesis and evaluation of novel Ozagrel-Paeonol codrug with antiplatelet aggregation activities as potent anti-stroke therapeutic agent**

Chijing Zuo^1#^, Fulong Yan^1#^, Jie Wang^1#^, Yulong Zhu^1^, Wenhui Luo^1^, Yan Liu^1^, Wanhui Liang^1^, Weidong Yu^1^, Jingwei Zhang^1^, Daiyin Peng^1,4^*, Xiaodong Ma^1*^, Can Peng^1,2,3,4,5,6^*

^1^School of Pharmacy, Anhui University of Chinese Medicine, Hefei 230012, China

^2^Generic Technology Research center for Anhui TCM Industry, Anhui University of Chinese Medicine, Hefei 230012, China

^3^Rural Revitalization Collaborative Technical Service Center of Anhui Province, Anhui University of Chinese Medicine, Hefei 230012, China

^4^MOE-Anhui Joint Collaborative Innovation Center for Quality Improvement of Anhui Genuine Chinese Medicinal Materials, Hefei 230012, China

^5^Anhui Province Key Laboratory of Pharmaceutical Preparation Technology and Application, Hefei, Anhui, 230012, China

^6^Center for Xin'an Medicine and Modernization of Traditional Chinese Medicine of IHM, Anhui University of Chinese Medicine, Hefei 230012, China

*Corresponding author:

Permanent address: College of Pharmacy, Anhui University of Chinese Medicine, 230012 Hefei, China.

Daiyin Peng, Email: pengdaiyin@163.com. Tel and Fax: +86-551-68129007

Xiaodong Ma, Email: o-omaxiaodong@163.com. Tel and Fax: +86-551-68129115

Can Peng, Email: pengcan@ahtcm.edu.cn. Tel and Fax: +86-551-68129115


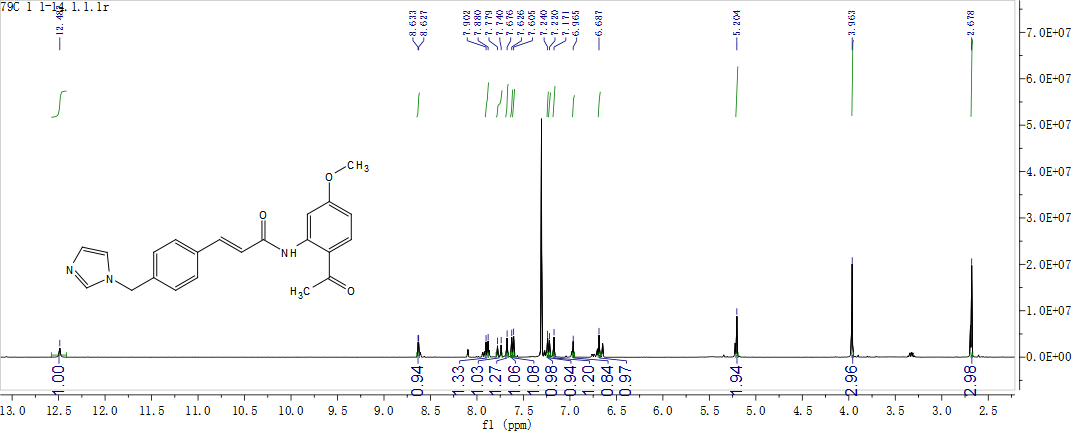


**Figure S1:** ^1^H NMR spectrum of **PNC_1_(3).**

**
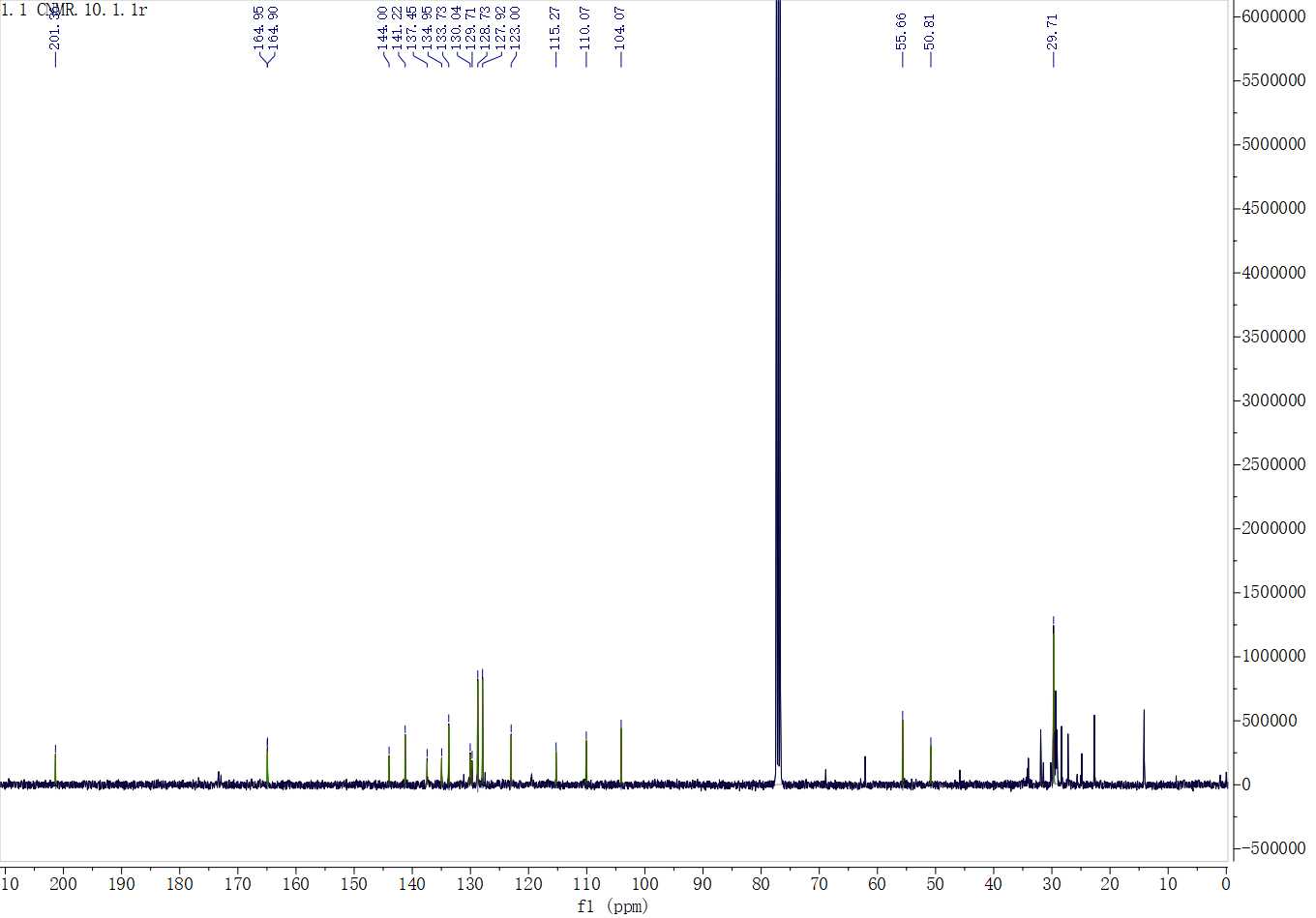
**

**Figure S2:** ^13^C NMR spectrum of **PNC_1_(3).**

**
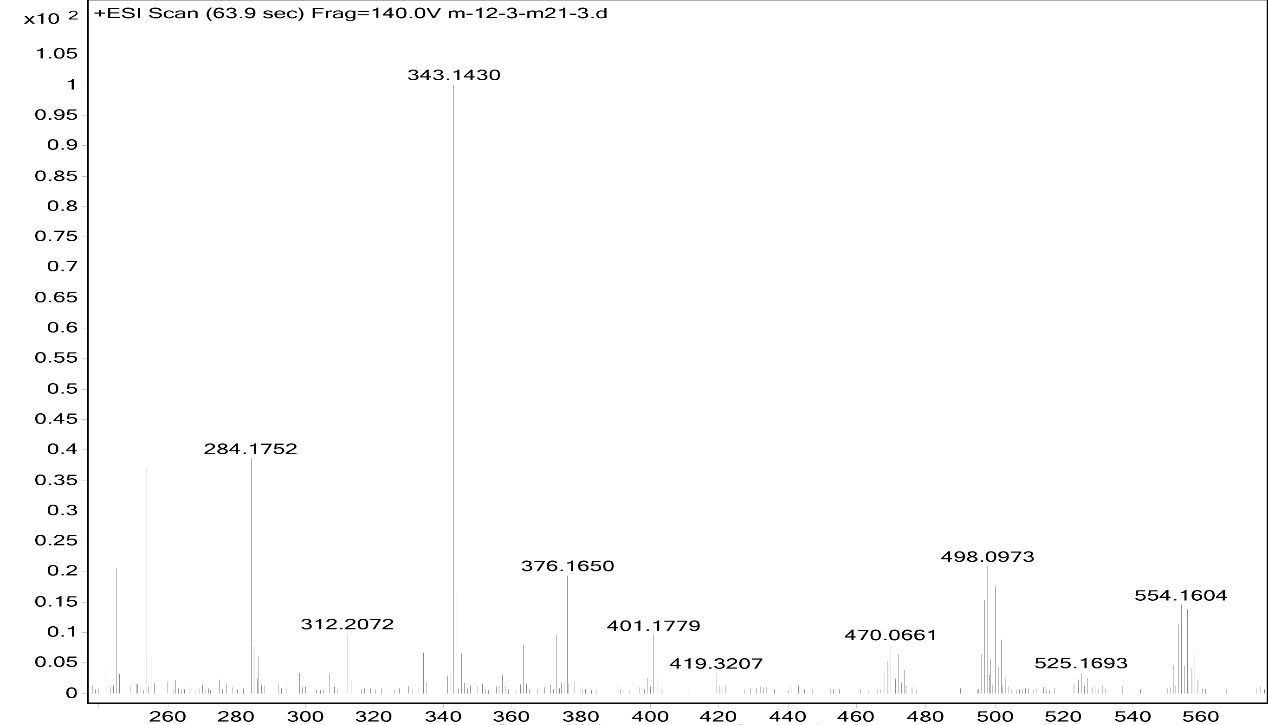
**

**Figure S3:** HR MS spectrum of **PNC_1_(3).**


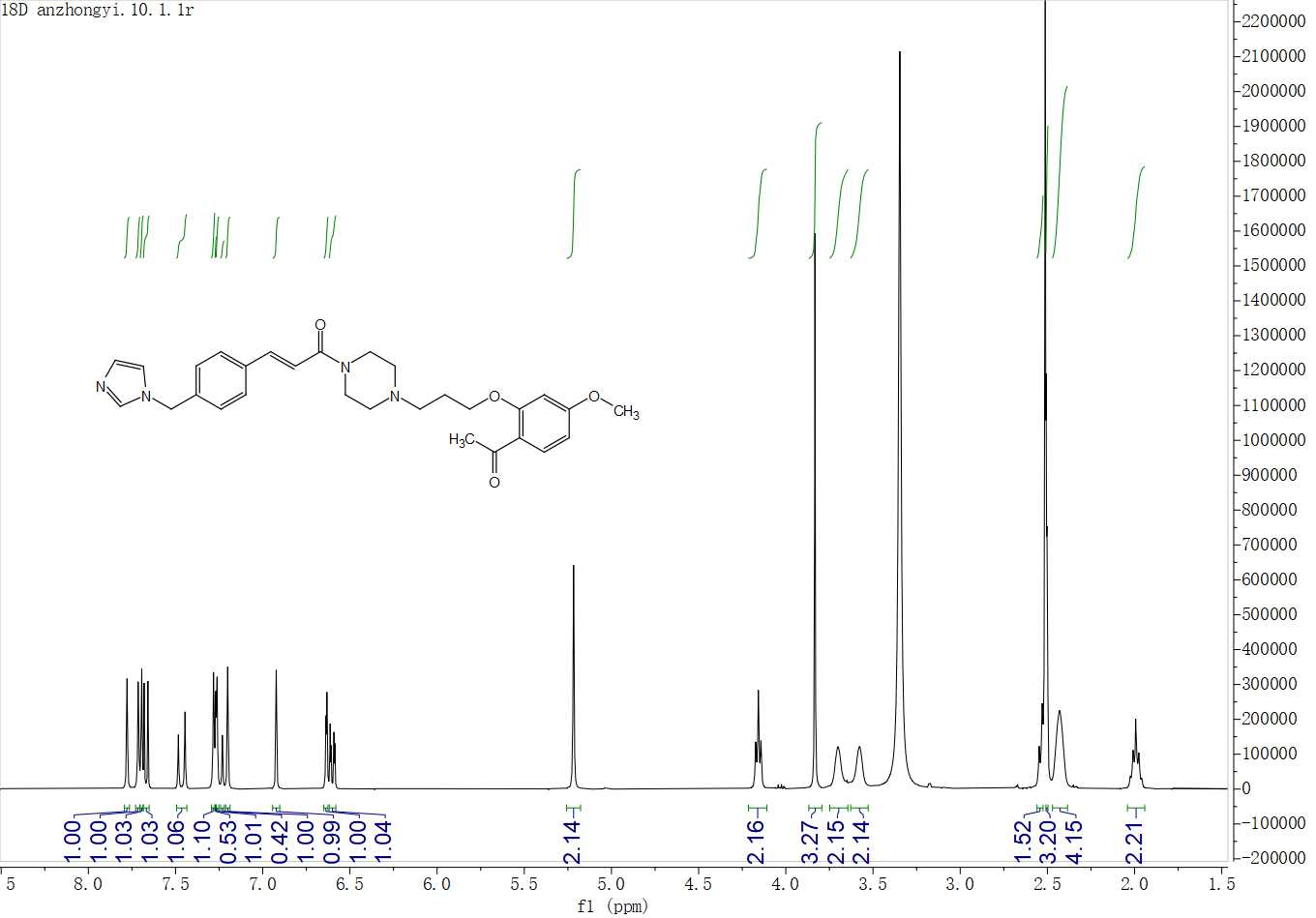


**Figure S4:** ^1^H NMR spectrum of **PNC_2_(8a).**

**
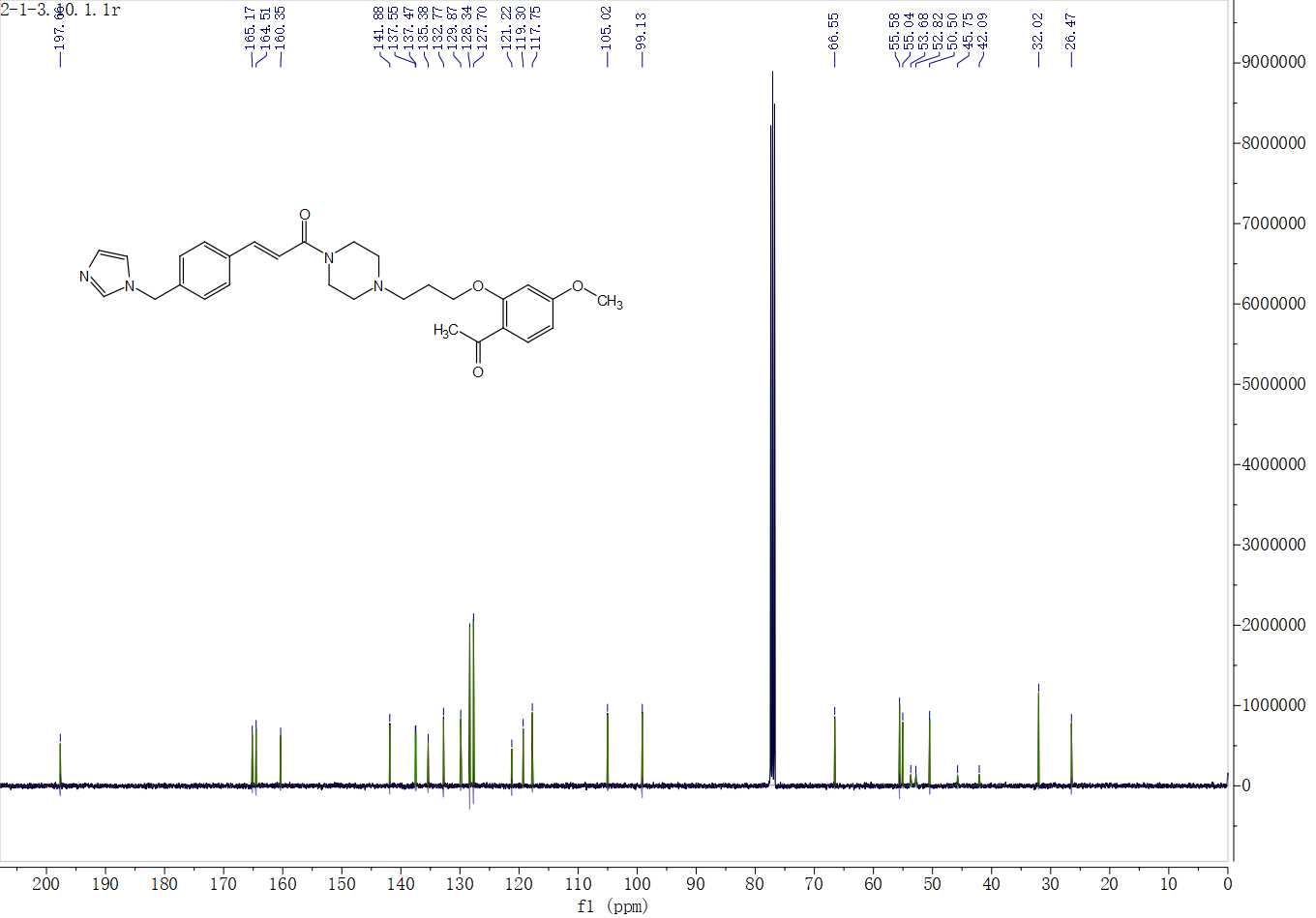
**

**Figure S5:** ^13^C NMR spectrum of **PNC_2_(8a).**

**
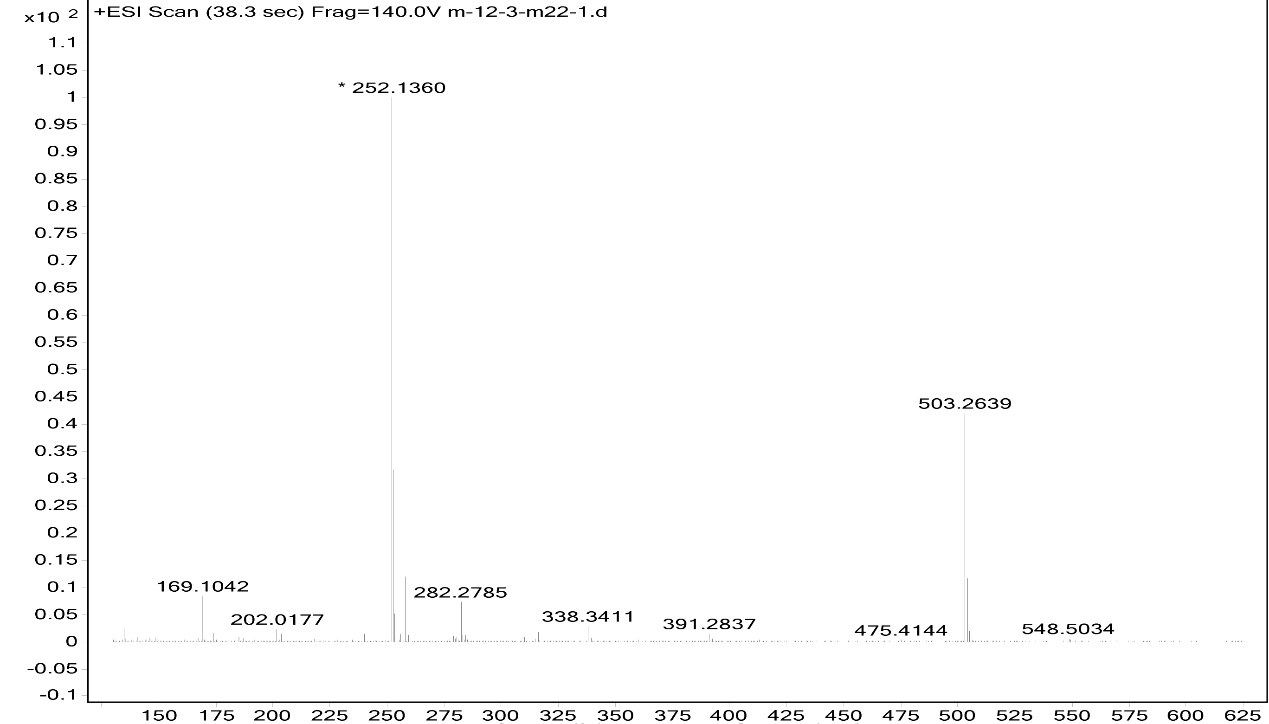
**

**Figure S6:** HR MS spectrum of **PNC_2_(8a).**


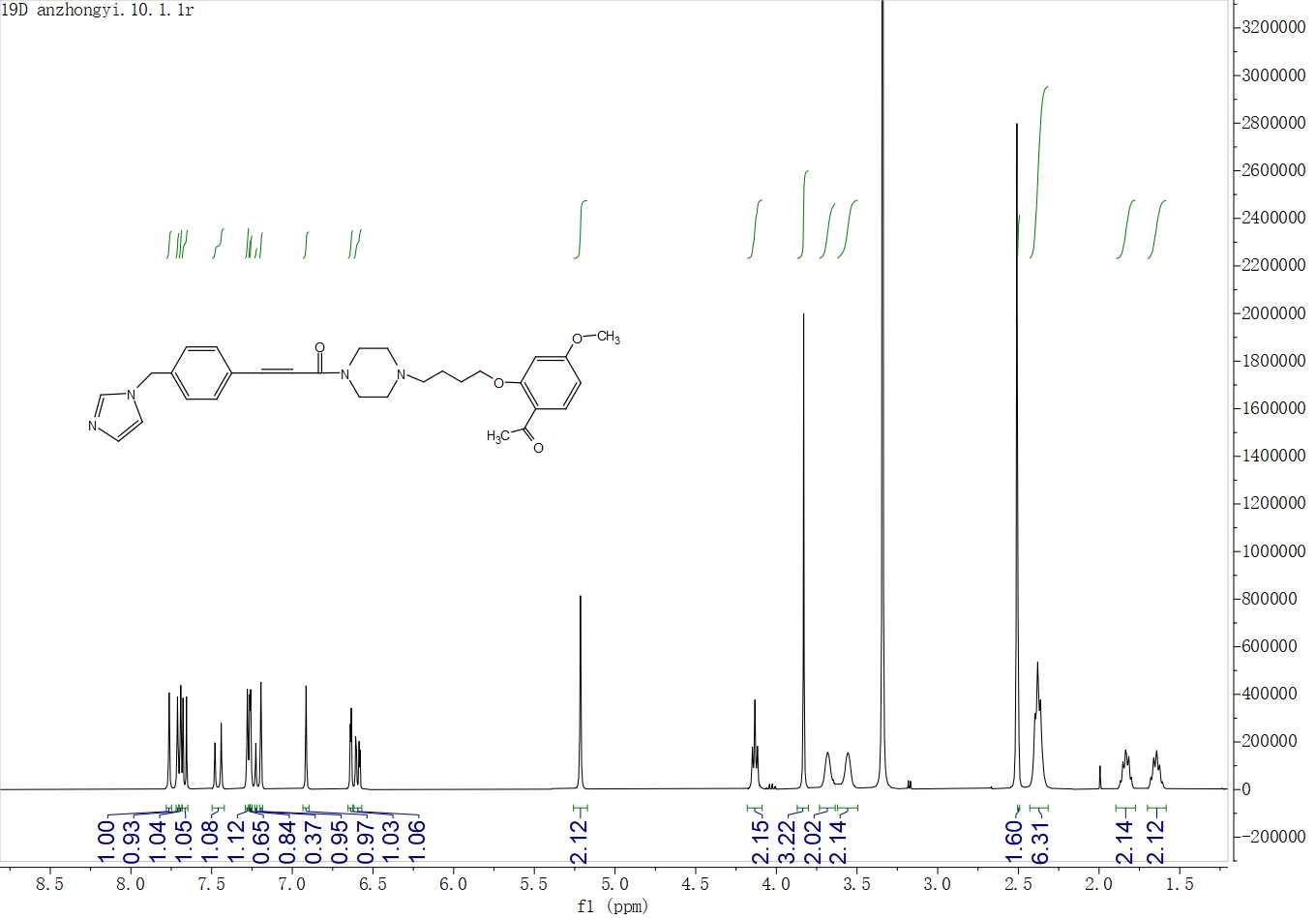


**Figure S7:** ^1^H NMR spectrum of **PNC_3_(8b).**

**
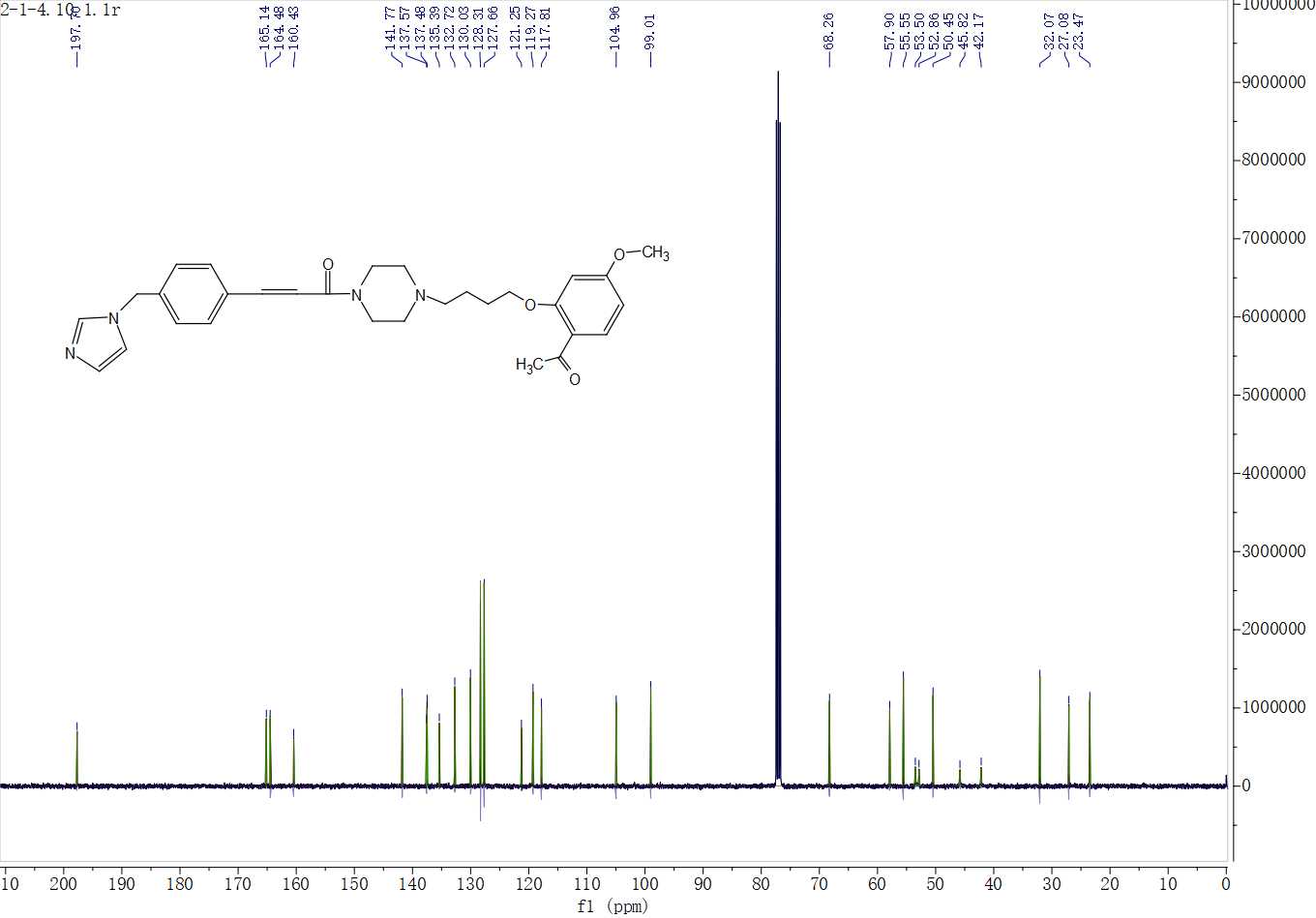
**

**Figure S8:** ^13^C NMR spectrum of **PNC_3_(8b).**

**
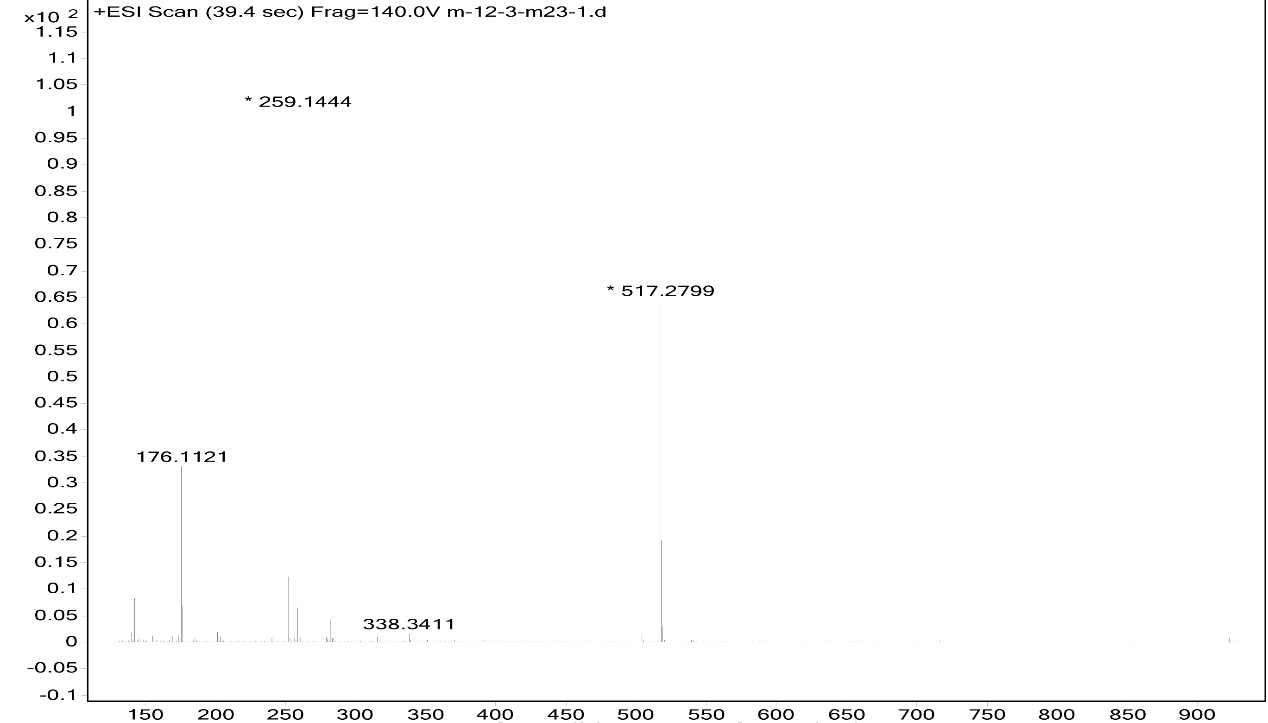
**

**Figure S9:** HR MS spectrum of **PNC_3_(8b).**


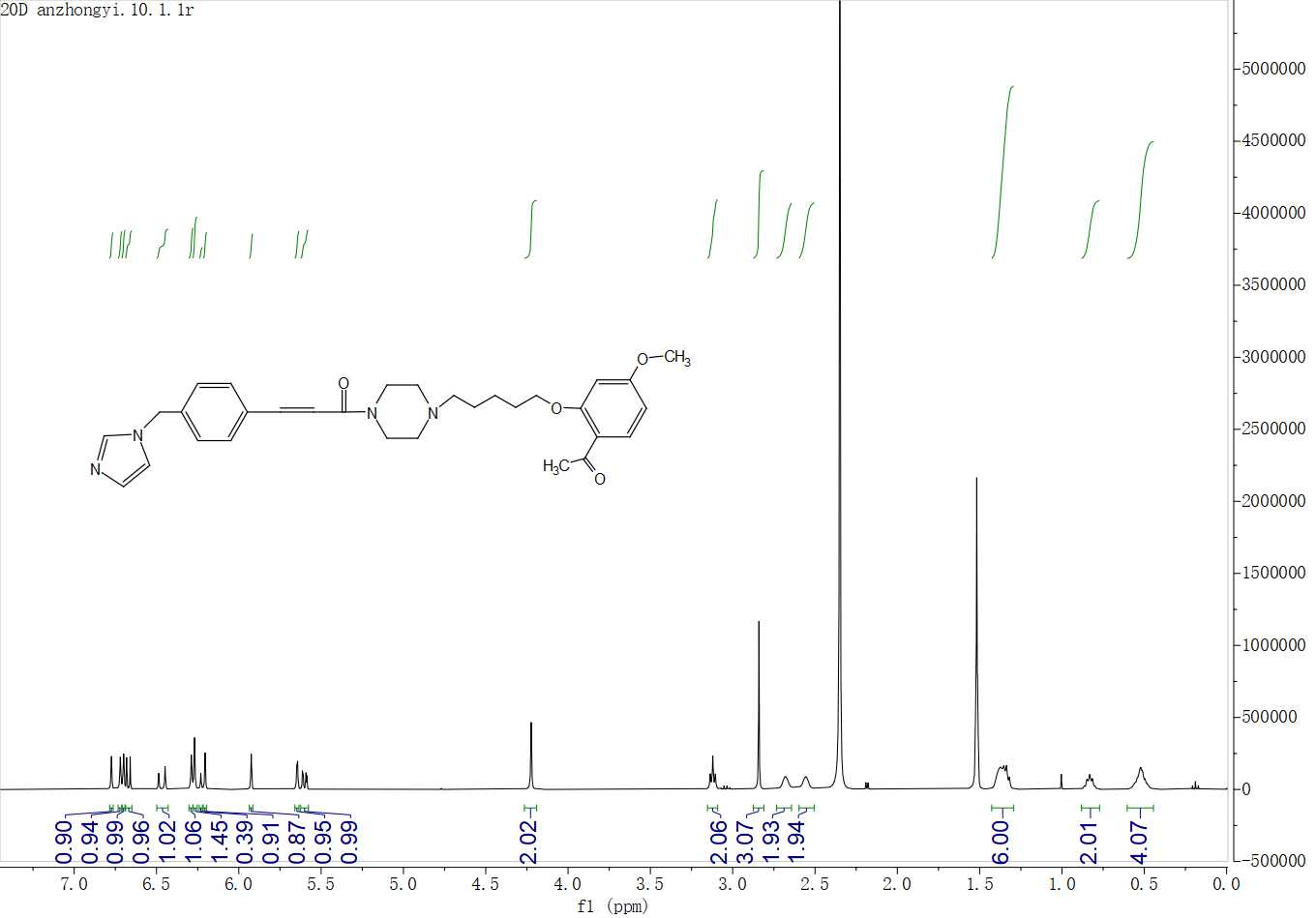


**Figure S10:** ^1^H NMR spectrum of **PNC_4_(8c).**

**
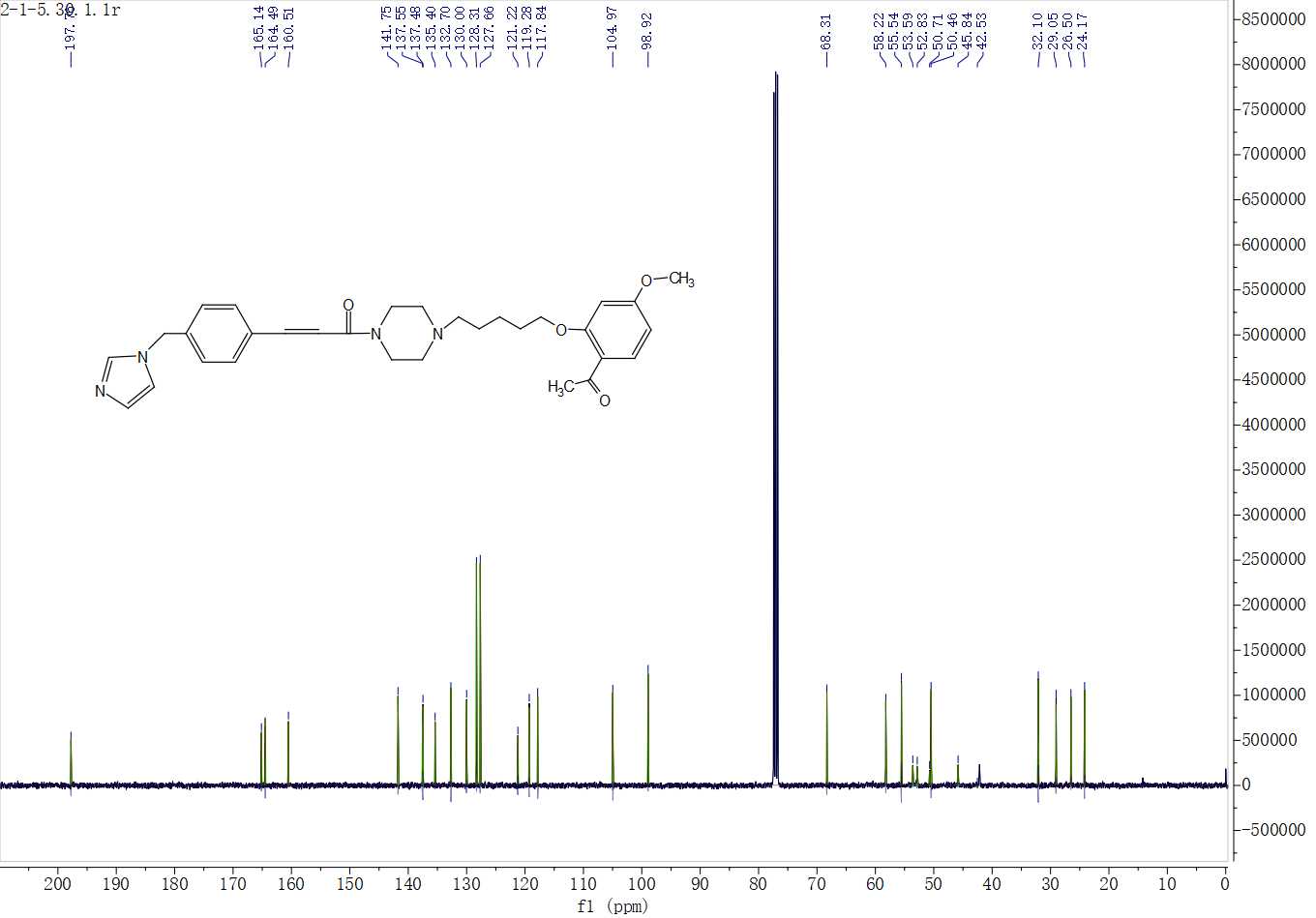
**

**Figure S11:** ^13^C NMR spectrum of **PNC_4_(8c).**

**
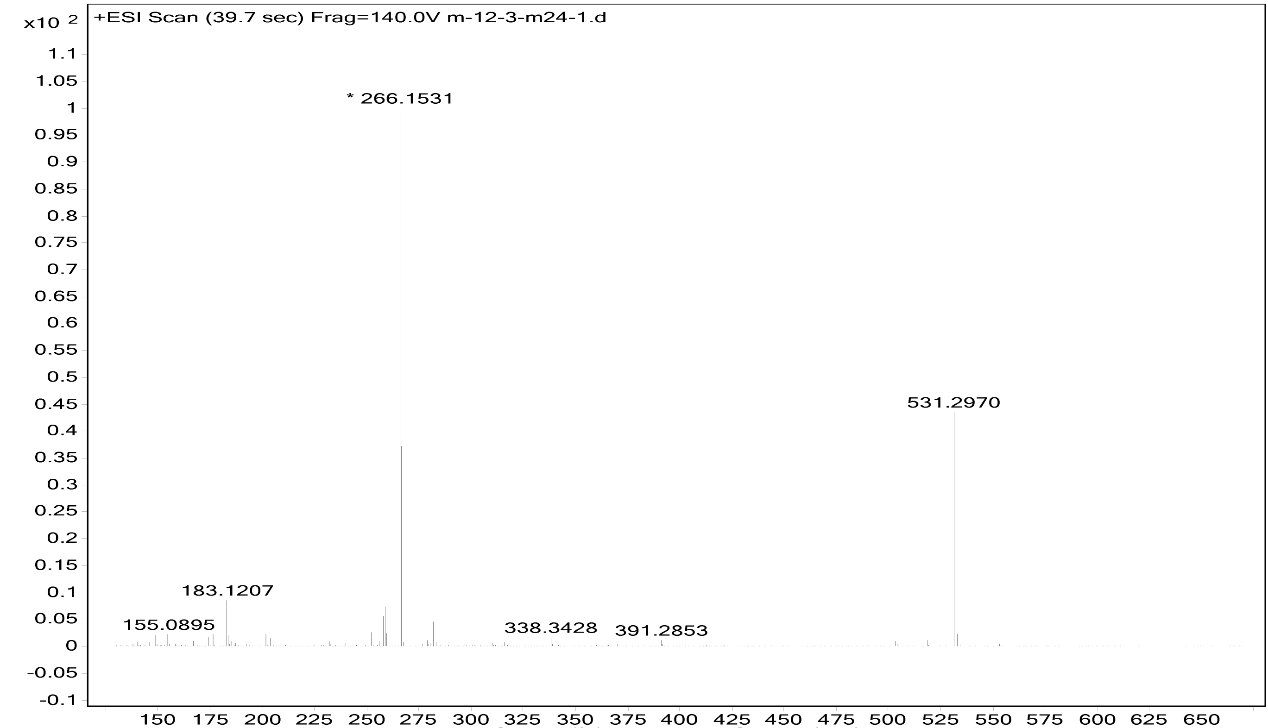
**

**Figure S12:** HR MS spectrum of **PNC_4_(8c).**


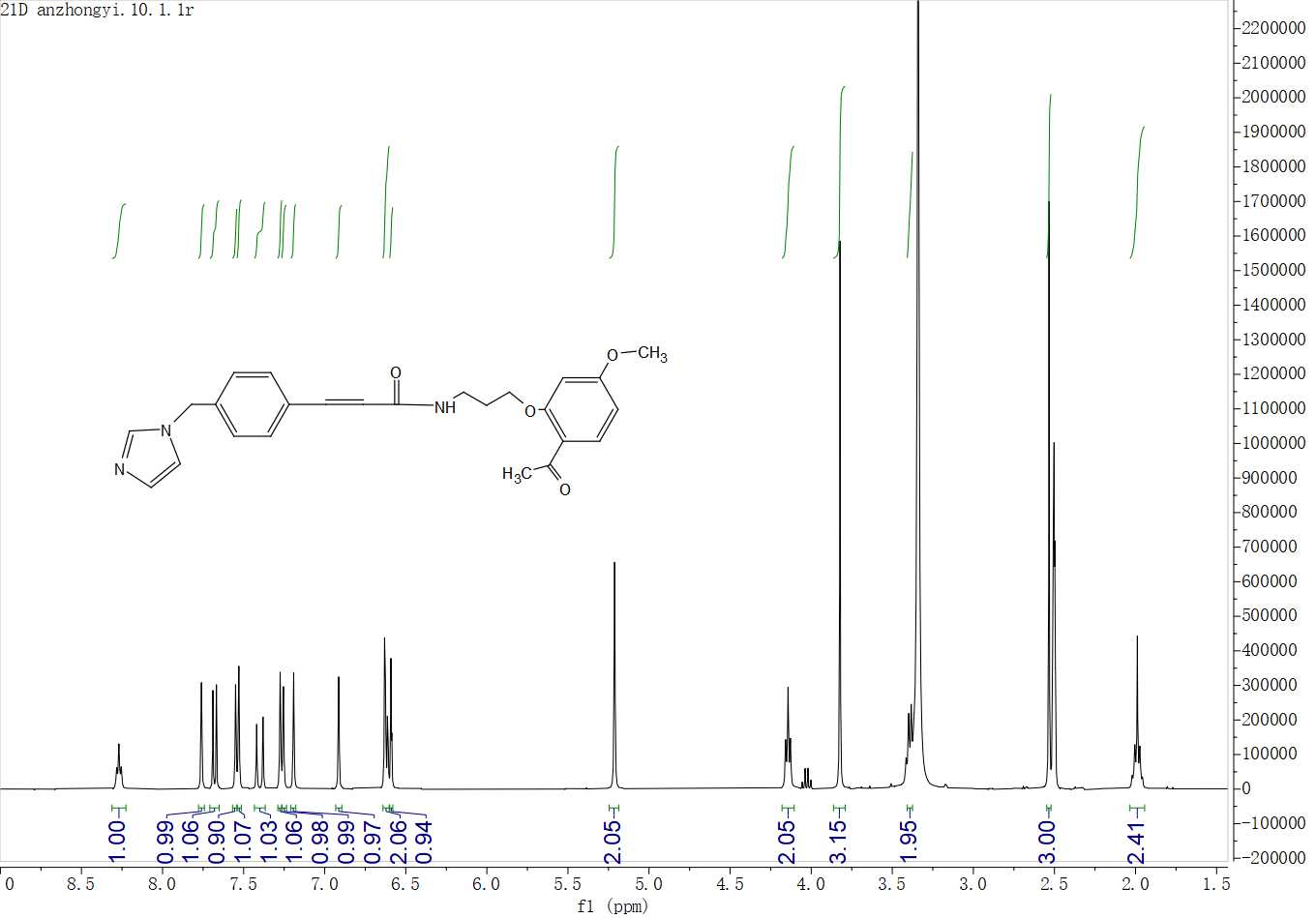


**Figure S13:** ^1^H NMR spectrum of **PNC_5_(11).**


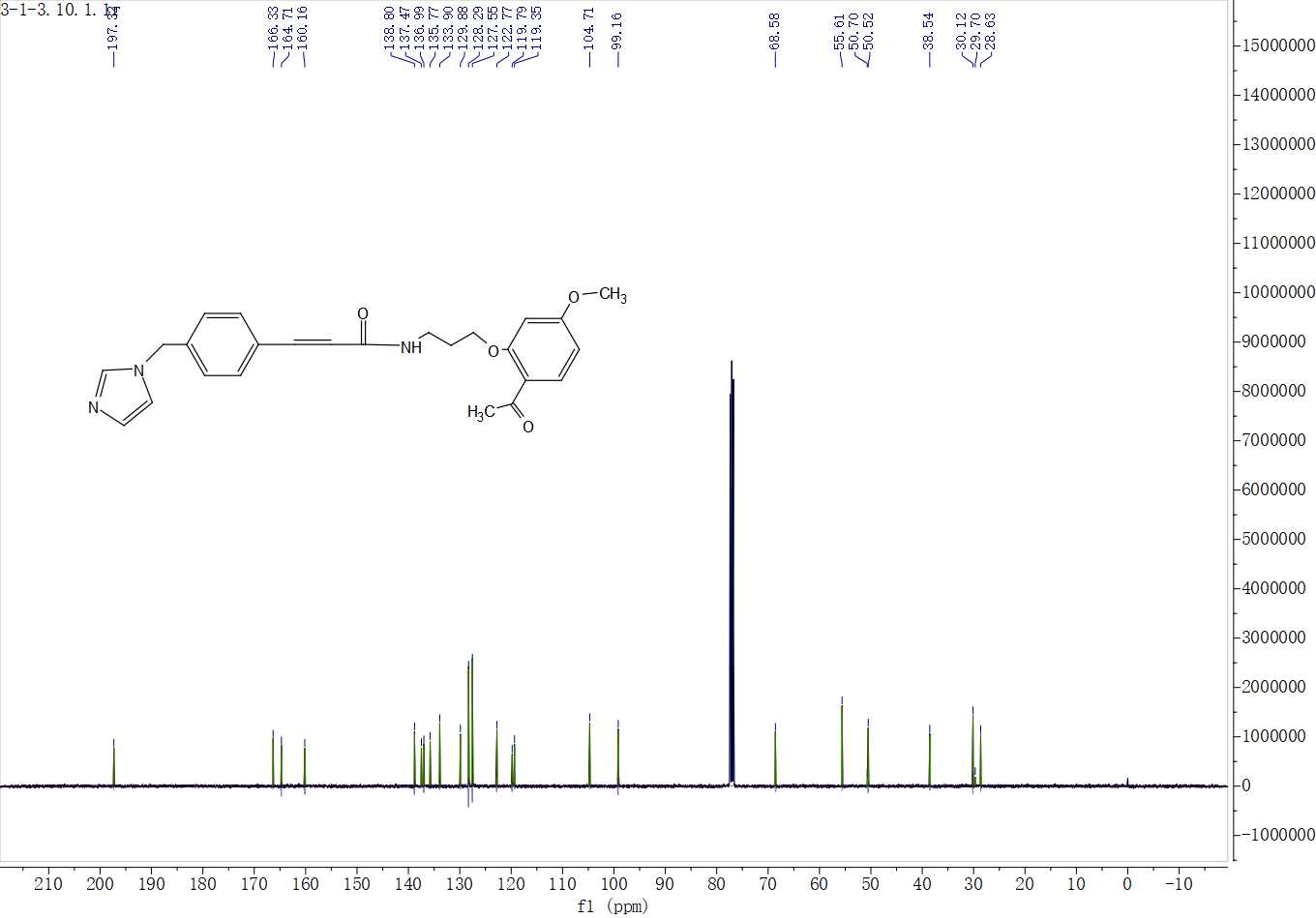


**Figure S14:** ^13^C NMR spectrum of **PNC_5_(11).**


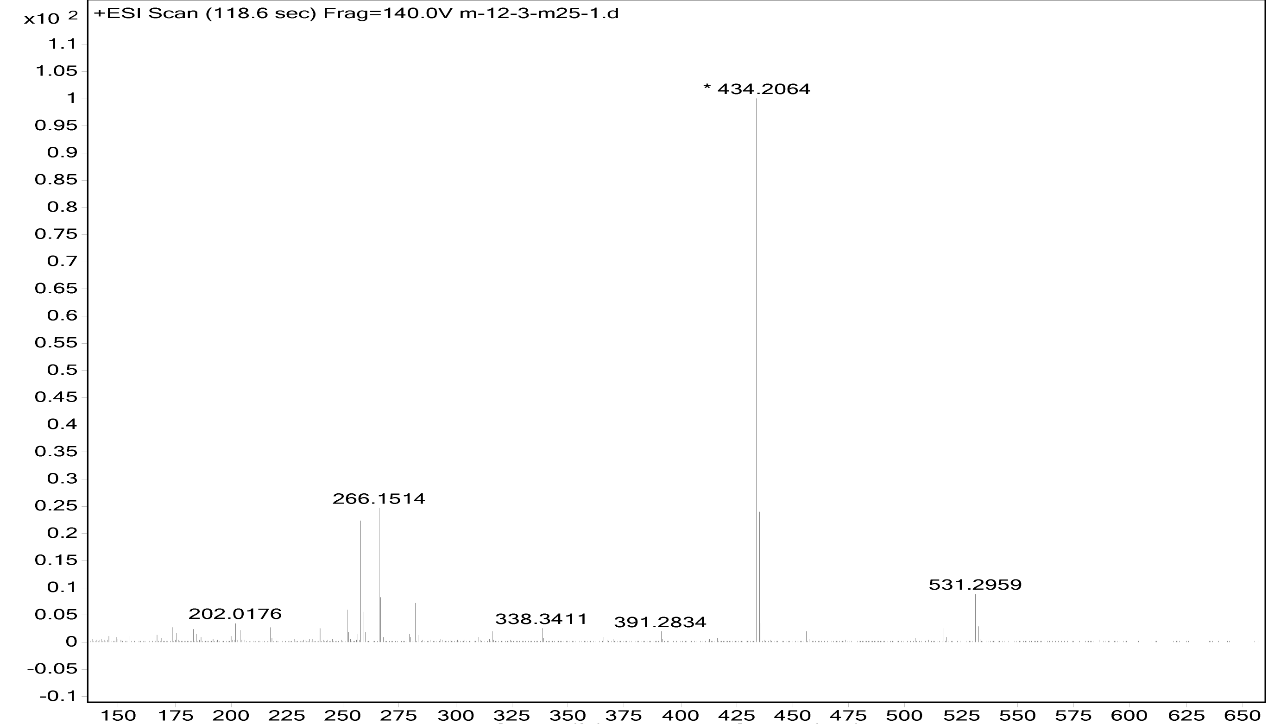


**Figure S15:** HR MS spectrum of **PNC_5_(11).**
